# Fuel-driven macromolecular coacervation in complex coacervate core micelles†

**DOI:** 10.1039/d2sc00805j

**Published:** 2022-03-31

**Authors:** Reece W. Lewis, Benjamin Klemm, Mariano Macchione, Rienk Eelkema

**Affiliations:** Department of Chemical Engineering, Delft University of Technology Van der Maasweg 9 2629 HZ Delft The Netherlands R.Eelkema@tudelft.nl

## Abstract

Fuel-driven macromolecular coacervation is an entry into the transient formation of highly charged, responsive material phases. In this work, we used a chemical reaction network (CRN) to drive the coacervation of macromolecular species readily produced using radical polymerisation methods. The CRN enables transient quaternization of tertiary amine substrates, driven by the conversion of electron deficient allyl acetates and thiol or amine nucleophiles. By incorporating tertiary amine functionality into block copolymers, we demonstrate chemical triggered complex coacervate core micelle (C3M) assembly and disassembly. In contrast to most dynamic coacervate systems, this CRN operates at constant physiological pH without the need for complex biomolecules. By varying the allyl acetate fuel, deactivating nucleophile and reagent ratios, we achieved both sequential signal-induced C3M (dis)assembly, as well as transient non-equilibrium (dis)assembly. We expect that timed and signal-responsive control over coacervate phase formation at physiological pH will find application in nucleic acid delivery, nano reactors and protocell research.

## Abstract 2

We apply an allyl acetate fuelled chemical reaction network (CRN) to control the coacervation of macromolecular species at constant physiological pH without the need for complex biomolecules.

## Graphical abstract

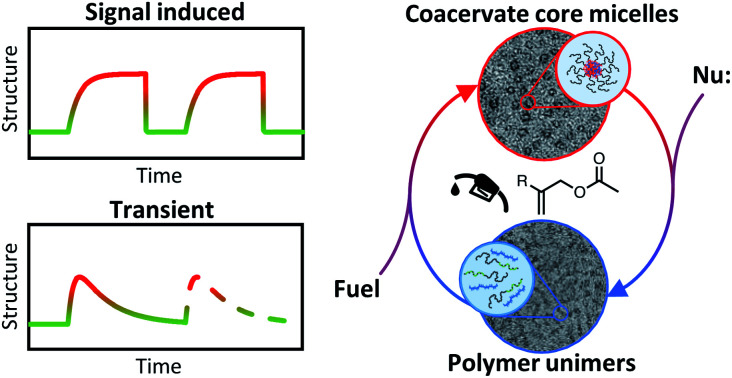

## Introduction

Dynamic control over assembly and disassembly allows for materials which respond to changes in their environment, with application in diagnostics,^1^ artificial protocell research,^2^ self-healing and drug release to name a few.^3–5^ Many synthetic materials have been designed to switch between equilibrium states upon reaction with a desired signal, for example micelles transitioning from assembled to disassembled states for site specific cargo release.^6^ In contrast to this, nature often utilises fuel-driven processes which achieve non-equilibrium structures with features including spatiotemporal control, responsiveness and autoconfiguration. These features allow for complex cellular functions such as division, motility and intracellular transport.^7,8^ Synthetic non-equilibrium assemblies can be attained from building blocks which are incorporated into a chemical reaction network (CRN). Here, non-assembling precursors are converted to an assembling product by irreversible reaction with a chemical reagent, the fuel. A second deactivating reaction later converts the product back to its non-assembling state. Thus, assembly only occurs where and when fuel is available, allowing for spatiotemporal control without an external change to the environment.^9^

In this work we describe a new approach for both signal-induced and fuel-driven coacervation of polyelectrolytes and explore its application in programmed micelle (dis)assembly. Coacervation occurs during the mixing of oppositely charged polyelectrolytes in aqueous solution, leading to the formation of a water-insoluble macromolecule rich phase (the complex coacervate) and a diluted bulk phase.^10,11^ When one (or more) water-soluble neutral block(s) are attached to the polyelectrolytes, complex coacervate core micelles (C3Ms) (also known as polyion complex micelles) can be formed.^12^ With a water-insoluble (but hydrated) coacervate core, C3Ms allow for incorporation and protection of hydrophilic substrates such as enzymes and nucleic acids, which is often not possible for conventional amphiphilic micelles.^13–17^

Coacervates are most favoured to form at low salt concentrations from large, fully ionized polyelectrolytes, combined at an equimolar ratio of cationic to anionic monomers.^18,19^ This causes inherent pH (for weak polyelectrolytes) and salt responsiveness in coacervates, inspiring the design of various stimuli responsive gels, encapsulated catalyst and drug delivery systems.^20–25^

For many biological and pH sensitive systems it is desirable to operate at constant pH, which in reversible coacervation is achieved using two general approaches. The first involves altering polyelectrolyte molecular weight, typically by enzymatic (de)polymerisation of nucleic acids and peptides.^26–28^ For example, the enzymatic polymerisation of uridine diphosphate (UDP) onto short RNA seeds has been exploited to trigger coacervate formation in the presence of spermine (a cationic polymer). This process can also be reversed in high phosphate buffer conditions, regenerating UDP and degrading the coacervate.^29,30^

A second approach is to change the ionisation or net charge of the polymer, which (at constant pH) is typified by chemical or enzymatic reactions on oligo-peptides.^31–34^ For example Donau *et al.* recently reported a fuel-driven system operating at pH 5.3, whereby an aspartate functionalised peptide is ring closed to the anhydride after addition of EDC (fuel), causing a change in peptide net charge from +1 to +3. This change in charge was sufficient to trigger the formation of a coacervate phase with RNA and, since the anhydride is unstable and hydrolyses back to the aspartate, the coacervate is transient.^32^

A common limitation of these constant pH systems is the requirement of specific biomolecules, complicating development and optimisation of fuel-driven coacervate systems. With a new CRN recently reported by our group,^35^ we envisaged a more general system utilising tertiary amine functionalised polymers which can be prepared by controlled radical polymerisation. This allows for simple, scalable synthesis of these materials with controllable architectures, which we exploit to achieve fuel-driven macromolecular coacervation within the context of C3Ms.

**Fig. 1:**
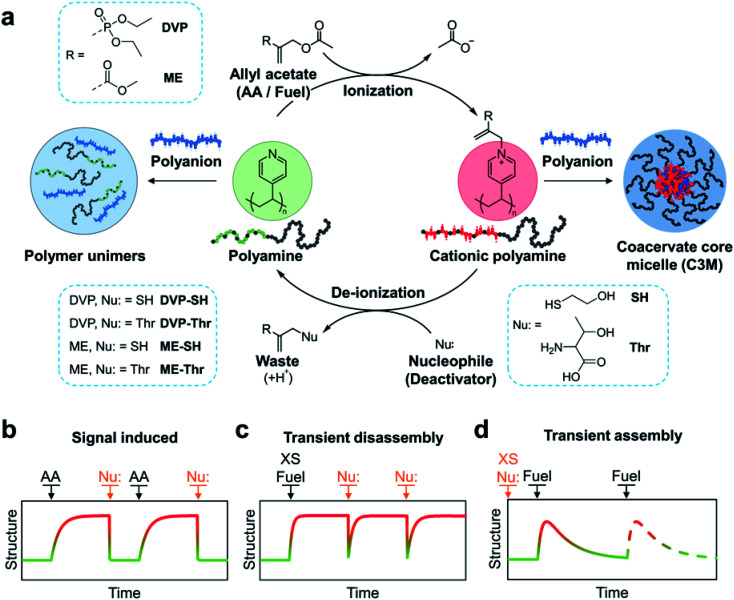
(a) Chemical reaction network (CRN) for reversible ionization of polyamines leading to controlled coacervation within complex coacervate core micelles (C3Ms). Specifically, tertiary amine functionalised block copolymers undergo a nucleophilic substitution reaction with electron deficient allyl acetates (AA), resulting in the formation of a cationic polyamine complex. A competing nucleophile (deactivator) can then participate in a second substitution reaction with the polyamine complex, regenerating the starting tertiary amine polymer and producing an AA-nucleophile waste product. When this cycle is conducted in the presence of suitable polyanions, polymer unimer to C3M transitions are obtained. (b) Graphical representation of the CRN operated with sequential equivalent additions of AA and nucleophile acting as signals for assembly and disassembly, respectively. (c) CRN operated with a starting excess of fuel and subsequent additions of nucleophile to trigger transient disassembly. (d) CRN operated with a starting excess of nucleophile and subsequent fuel additions to trigger transient assembly.

The CRN is fuelled by electron deficient allyl acetates (AA), which when reacted with tertiary amines yield a quaternary cationic adduct. Reaction of this adduct with a competing nucleophile is then able to regenerate the starting tertiary amine, completing the cycle. In this work we demonstrate the utility of the allyl acetate CRN to achieve reversible coacervation within C3Ms by transient quaternization of tertiary amine functionalised block copolymers in the presence of a polyanion (Fig. 1a). We first studied sequential equivalent additions of AA and nucleophile which act as signals to transition between equilibrium micelle assemblies and a unimer mixture solution (Fig. 1b). We then examined systems where both the ionization and deionization reactions compete, resulting in non-equilibrium structures which are kinetically controlled. In these scenarios AA acts as a fuel. By starting with an excess of fuel (AA), subsequent nucleophile additions can trigger transient C3M disassembly (Fig. 1c). Conversely, starting with an excess of a weak nucleophile, fuel (AA) additions can trigger transient C3M assembly (Fig. 1d).

## Results and discussion

### Assembly and morphology of C3Ms

**Fig. 2:**
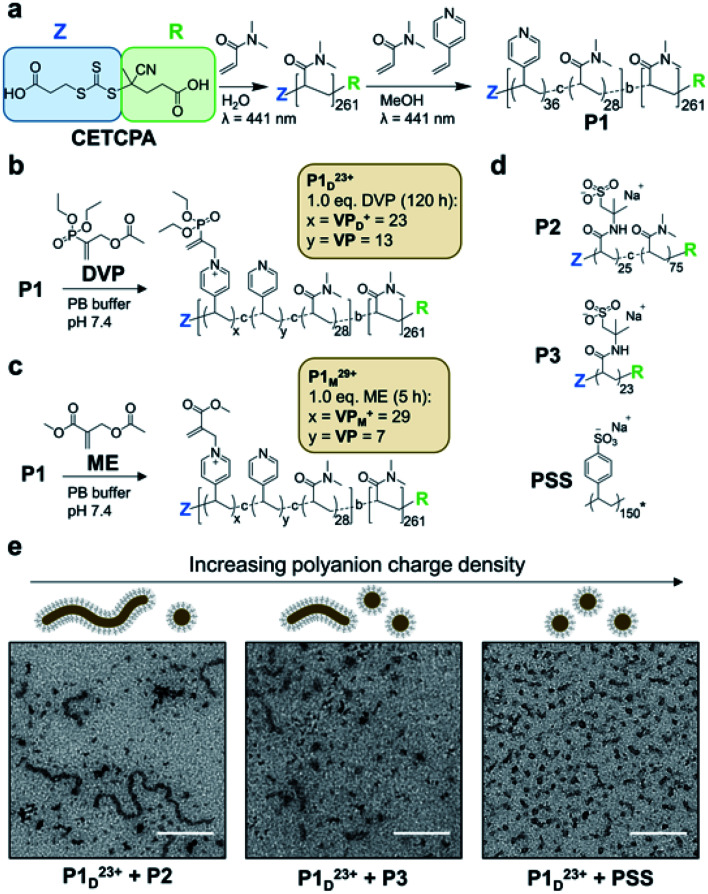
Polyamine block copolymer (P1) synthesis and assembly into C3Ms. (a) Two step synthesis of P1 from RAFT polymerisation of DMA and 4VP with chain transfer agent (4-((((2-carboxyethyl)thio)carbonothioyl)thio)-4-cyanopentanoic acid, CETCPA). (b) Conversion of neutral VP units to cationic VP_D_^+^ in P1 (20 mM) by reaction with DVP (1.0 eq.) in pH 7.4 PB buffer (100 mM) yielding P1_D_^23+^. (c) Conversion of neutral VP to cationic VP_M_^+^ in P1 (20 mM) by reaction with ME (1.0 eq.) in pH 7.4 PB buffer (100 mM) yielding P1_M_^29+^. Acetate counterions in P1_D_^23+^ and P1_M_^29+^ omitted for visual clarity. (d) Structures of polyanions combined P1_D_^23+^ during initial micelle assembly studies. P2 and P3 were synthesised by RAFT polymerisation of 2-acrylamido-2-methylpropane sulfonic acid (AMPS) and DMA with CETCPA as the chain transfer agent. Poly(sodium 4-styrenesulfonate) (PSS) was commercially obtained with a reported MW of 200 kDa, while *M*_*n*,GPC_ = 31.5 kDa was measured in our laboratory. *The average degree of polymerisation (150) as shown in the figure is therefore a conservative estimate based on *M*_*n*,GPC_ to highlight its relatively high charge density. (e) TEM images and schematic illustration of coacervate core micelles (C3Ms) obtained from the combination of P1_D_^23+^ with polyanions of varied charge density at 4 mM amine and anionic functional group concentration (scale bar is 200 nm). Samples were visualised with uranyl acetate staining. Additional TEM, cyroTEM and DLS data are presented in Fig. S11–S12.†

To realise reversible coacervation within C3Ms induced by the allyl acetate CRN, we first synthesised a polyamine block copolymer (P1, Fig. 2a). This was prepared by a two-step reversible addition fragmentation chain-transfer (RAFT) process.^36^ First, dimethylacrylamide (DMA) was polymerised to form the water-soluble neutral block. This was then chain-extended by copolymerisation of 4-vinyl pyridine (4VP) and DMA to form the ionizable core block. A shift to lower retention times of a single GPC peak demonstrated successful chain extension, with the product P1 having a low dispersity (*Đ* = 1.20) and molecular weights determined by GPC and conversion in excellent agreement (*M*_*n*,GPC_ = 32.9 kDa, *M*_*n*,conv_ = 32.8 kDa). We selected pyridine as the tertiary amine functionality as it has a conjugate acid pKa of 5.2,^37^ ensuring that negligible protonated (cationic) species exist at pH 7.4 before addition of fuel. Water soluble DMA was incorporated into the ionizable core block to avert the formation of amphiphilic micelles in pH 7.4 aqueous solutions, which was observed to occur for polyamine block copolymers containing only 4VP in the core.

To operate the reaction cycle we studied two different AA molecules; diethyl(α-acetoxymethyl) vinylphosphonate (DVP)^35^ and methyl 2-(acetoxymethyl)acrylate (ME),^38^ the latter of which we found to be more reactive due to its stronger electron withdrawing R group. As an initial test we studied the AA-driven ionization of P1 by combining DVP (1.0 eq.) with an aqueous solution of P1 (1.0 eq. 4VP, 20 mM, pH 7.4). This resulted in approximately 65% ionization of amine functionality after 120 h (P1_D_^23+^, Fig. 2b). Considering an average incorporation of 36 4VP units per polymer chain for P1, this equates to approximately 23 cationic units (VP_D_^+^) and 13 neutral (VP) per chain. Repeating the experiment with ME resulted in greater amine ionization (80%) after only 5 h (P1_M_^29+^, Fig. 2c). Such conversion yielded an estimated 29 cationic pyridine units (VP_M_^+^) per chain. In both cases complete ionization was not observed, perhaps due to increasing steric hindrance as conversion increased.^39^ We next investigated whether P1_D_^23+^ could form C3Ms when mixed with a polyanion, without which both P1 and P1_D_^23+^ are near indistinguishable by dynamic light scattering (DLS) (Fig. S11a†). Considering the incomplete ionization, as well as the incorporation of neutral DMA in the core block, polyanion selection was likely to significantly influence C3M assembly. This is because a minimum polyelectrolyte length and degree of ionization, together referred to here as charge density is typically required for coacervate phase formation.^10,18,19,40,41^ Combining P1_D_^23+^ with various polyanions (Fig. 2d) at equal concentration of cationic to anionic functionality (4 mM) yielded C3Ms as demonstrated by increased number average diameter and light scatter through DLS (Fig. S11c†). Interestingly, TEM images demonstrated a morphological transition from a mixture of worm-like and spherical micelles to almost exclusively spherical micelles with increasing polyanion charge density (Fig. 2e). A commercially obtained 200 kDa poly(sodium 4-styrenesulfonate) (PSS) was selected for further study, as it formed relatively homogeneous spherical micelles with P1_D_^23+^. The PSS C3Ms were then further characterised by cryoTEM which confirmed the formation of 11 nm spherical species (Fig. S12b†).

### Signal-induced C3M (dis)assembly

**Fig. 3:**
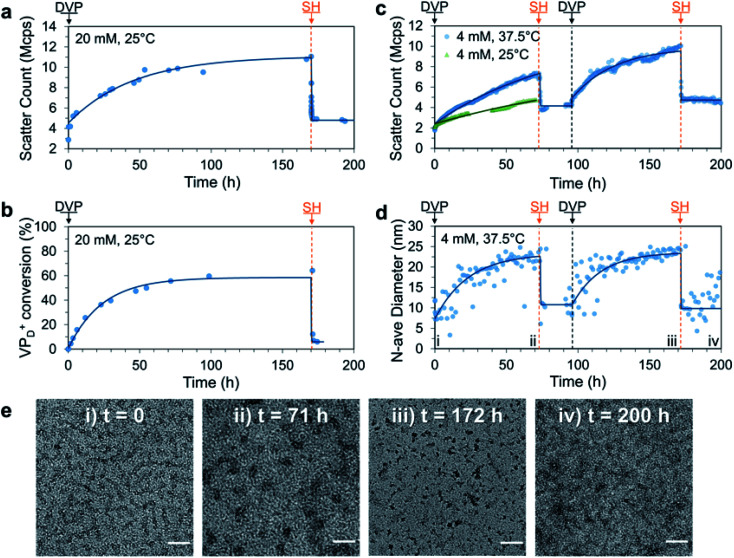
Signal-induced C3M (dis)assembly from sequential 1.0 eq. additions of DVP and SH to a mixture of P1 and PSS in 100 mM PB pH 7.4 buffer. Experiment was first conducted at 25 °C, 20 mM P1 and PSS with (a) change in scatter count observed by DLS and (b) matching conversion of VP to VP_D_^+^ in P1 monitored by ^1^H NMR (without PSS to avoid suppression of micelle core signals). The experiment was repeated at 37.5 °C, 4 mM and monitored by DLS and TEM. (c) Change in scatter count (70 h after first DVP addition is also shown for 25 °C) and (d) number average diameter over time, time points corresponding to TEM images marked with Roman numerals. (e) Representative TEM images taken throughout 37.5 °C, 4 mM cycle at: (i) *t* = 0 (unimers), (ii) *t* = 71 h (C3Ms), (iii) *t* = 172 h (C3Ms) and (iv) 200 h (unimers) with scale bars at 50 nm. Exponential curves drawn after each addition are to guide the eye. Additional DLS, ^1^H NMR and TEM data including full details of 4 mM, 25 °C experiment can be found in the Fig. S13–S17.†

Having established the formation of C3Ms by combination of P1_D_^23+^ and PSS, we next sought to achieve signal-induced C3M assembly as depicted in Fig. 1b. First, an aqueous buffered solution of P1 and PSS at equimolar amine and anionic functionality (20 mM, pH 7.4) was prepared and transferred to a cuvette for *in situ* analysis of C3M assembly by DLS. To this mixture, we added DVP (1.0 eq. *vs.* VP), resulting in an approximate 3-fold increase in the light scatter intensity (scatter count) over 170 h (Fig. 3a). In parallel, the experiment was performed without PSS and monitored by ^1^H NMR to determine the extent of conversion from neutral VP to cationic VP_D_^+^ in P1 (Fig. 3b). Note that PSS was excluded from the ^1^H NMR experiment to avoid suppression of the charged polyamine complex protons due to inclusion in the micelle core. The scatter count increase and conversion to VP_D_^+^ were found to closely correlate, indicating the successful assembly of micelles after *in situ* ionization of P1 with DVP. To complete the cycle and destroy the micelles, neutral VP needs to be regenerated by reaction of cationic VP_D_^+^ with a competing nucleophilic species. Preliminary experiments demonstrated that the rate of deionization strongly correlated with nucleophile nucleophilicity (R–SH > R_2_NH > RNH_2_), so for convenient observation 2-mercaptoethanol (SH, 1.0 eq.) was added to both the DLS and ^1^H NMR solutions at 170 h. This promptly caused significant regeneration of VP (VP_D_^+^ conversion reduced from 64% to 7% within 40 minutes) along with formation of the DVP-SH waste product. This nucleophile triggered deionization of quaternized polyamine complexes in P1 resulted in rapid C3M disassembly, as demonstrated by a reduction in DLS scatter count to near starting levels (from 11 to 5.5 Mcps in 5 minutes). Additionally, since this process results in regeneration of the starting polyamine, it theoretically can be repeatedly cycled. Indeed, further addition of DVP (1.0 eq.) to the NMR experiment did demonstrate reformation of VP_D_^+^ in P1 to similar conversion (69%, Fig. S14a†). However, no further DLS analysis was conducted since at the high polymer concentration (preferred for ^1^H NMR analysis), DLS object diameter data was found to be noisy and unreliable, perhaps due to multiple scattering effects (Fig. S14c†).

To improve the quality of DLS data collected, we repeated the experiment at reduced polymer concentration (4 mM) and analysed it by DLS and TEM. This was conducted at both 25 °C (like all other experiments) and 37.5 °C to investigate if this cycle could operate under biologically relevant conditions. After addition of DVP to both samples, the scatter count increased significantly faster at 37.5 °C than at 25 °C, with a respective doubling time of 12 and 56 h (Fig. 3c). This demonstrated that mild heating can accelerate the reaction between DVP and the tertiary amine substrate, and as such the 37.5 °C condition was selected for convenient study of two complete reaction cycles (full data from single cycle at 25 °C in Fig. S15†).

To achieve two signal-induced cycles, sequential additions of DVP and SH were twice applied to a single solution of P1 and PSS (4 mM). Like the previous data collected at 20 mM, an increase in scatter count was observed after each addition of DVP, while each addition of SH gave a sharp decrease (Fig. 3c). The DLS scatter count before and after addition of SH was found to be higher in the second cycle (from 10.0 to 4.6 Mcps) compared to the first (from 7.0 to 3.7 Mcps). This may be due to an incomplete first cycle reaction, waste accumulation or slight evaporation of aqueous solvent during the heated experiment (cuvette was parafilm covered). Importantly, an increase in the number average diameter from approximately 10 to 25 nm after addition of DVP was observed in both cycles, with thiol additions reverting the diameter back to the starting point (Fig. 3d). Such a change in number average size indicates a transition from unimers (10 nm) to spherical micelles (25 nm), an explanation further supported by TEM images taken at the end of each addition. Here, corresponding transitions from faint wormlike species to darker spherical objects of 23.7 ± 3.6 nm and 14.0 ± 3.4 nm were observed following the first and second DVP addition, respectively (Fig. 3e). Combined these data demonstrate signal-induced assembly and disassembly of C3Ms after addition of DVP and SH, respectively.

**Fig. 4:**
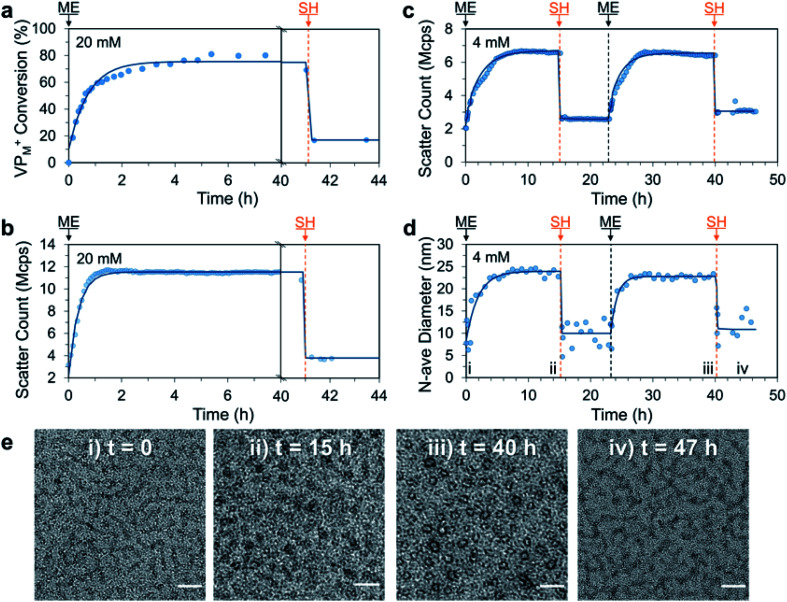
Signal-induced C3M (dis)assembly from sequential 1.0 eq. additions of ME and SH to a mixture of P1 and PSS in 100 mM PB pH 7.4 buffer at 25 °C. Experiment was first conducted at 20 mM P1 and PSS with (a) conversion of VP to VP_M_^+^ in P1 monitored by ^1^H NMR (without PSS to avoid suppression of micelle core signals) and (b) matching DLS scatter count data. The experiment was repeated at 4 mM and monitored by DLS and TEM. (c) Change in scatter count and (d) number average diameter over time (time points corresponding to TEM images marked with Roman numerals). (e) Representative TEM images taken throughout 4 mM cycle at: (i) *t* = 0 (unimers), (ii) *t* = 15 h (C3Ms), (iii) *t* = 40 h (C3Ms) and (iv) 47 h (unimers). Scale bars 50 nm. Exponential curves drawn after each addition are to guide the eye. Additional DLS, ^1^H NMR and TEM data can be found in the Fig. S18–S24.†

With this promising result we decided to investigate if ME, a more reactive AA could be utilised to achieve the signal-induced C3M switching behaviour in an accelerated fashion. In this case, addition of ME (1.0 eq. *vs.* VP) to a 20 mM aqueous solution of P1 and PSS resulted in a similar 3-fold increase in DLS scatter count over only 2 h. This was in agreement with ^1^H NMR measurements (without PSS), which indicated approximately 60% conversion of VP to VP_M_^+^ after 2 h and a peak conversion of 80% after 5 h (Fig. 4a and b). Interestingly the scatter count remained constant after 1.5 h, which indicates a critical conversion near 60%, beyond which little change in the extent of micelle assembly is observed. Also of note, is the slight decrease in NMR conversion from 5 to 40 h (80% to 70%), which we attribute to phosphate nucleophilic substitution on the VP_M_^+^ quaternary complex (phosphate from the PB buffer, Fig. S18†). Addition of SH (1.0 eq.) then rapidly (within 10 minutes) regenerated neutral VP (conversion to VP_M_^+^ reduced from 70% to 20%) and yielded the ME-SH waste product. Like the DVP based system, this triggered reduction in ionization of P1 appears to result in C3M destruction as evidenced by a reduction in scatter count from 11.3 to 4.6 Mcps. Re-formation of VP_M_^+^ in a second cycle was found to proceed to a similar conversion (78%) 4 h after a further addition of ME (1.0 eq., Fig. S21a†), indicating ability for use in multiple cycles. NMR experiments also identified the formation of a double Michael adduct (ME-2SH, two additions of SH to ME), which formed in appreciable quantity when an excess of SH was added (Fig. S18–S19†).

We then conducted a complete two cycle experiment analysed by DLS at 4 mM polymer concentration. As expected, each addition of ME (1.0 eq.) led to an increase in scatter count over 7 h (slower rate than at 20 mM due to dilution), while each addition of SH (1.0 eq.) gave a sharp decrease (Fig. 4c). Promisingly, the peak and minimum scatter counts after ME and SH addition were found to be similar for each cycle (within 15%), indicating excellent repeatability of the reaction cycle for C3M assembly. A shift in number average diameter from approximately 10 to 25 nm after addition of ME was observed, which reverted to approximately 10 nm after addition of SH (Fig. 4d), matching the endpoints observed with DVP. TEM images taken at the end of each addition showed transitions from faint wormlike species to darker spherical objects of 23.3 ± 4.4 nm and 17.8 ± 2.6 nm after the first and second ME addition, respectively (Fig. 4e). Together, these results demonstrate ME signal-induced assembly of C3Ms in a similar but accelerated manner to that observed with DVP.

### Transient C3M disassembly

**Fig. 5:**
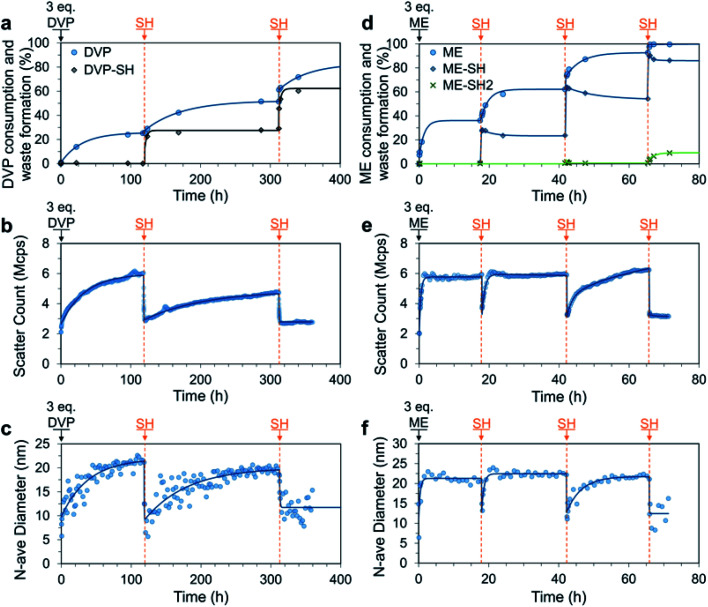
Transient C3M disassembly triggered by sequential 1.0 eq. additions of SH to a mixture of P1 and PSS (4 mM), where excess (3.0 eq.) DVP (a–c) or ME (d–f) was added at *t* = 0 to pre-form the micelles. All experiments were conducted in 100 mM PB pH 7.4 buffer. The process was monitored by ^1^H NMR (a and d), where only allyl acetate (AA) consumption and waste formation was quantified since PSS was included in the ^1^H NMR experiment, masking the micelle core signals. C3M assembly was confirmed by DLS measurements quantitating the scatter count (b and e) and number average diameter (c and f). Exponential curves drawn after each addition are to guide the eye. Additional DLS, ^1^H NMR and TEM data can be found in Fig. S25–S29.†

We next studied the response of SH additions to a system containing an excess of AA, thereby allowing kinetics to control the assembly and disassembly processes and the composition of the mixture at any given time (Fig. 1c). To this end, 3.0 equivalents of DVP were added to a solution of P1 and PSS (both 4 mM). As expected, we observed a slow but strong increase in the DLS scatter count and number average size, with the number average size plateauing after 100 h (increase from approximately 10 to 22 nm). Over the same time period 25% of the available DVP was consumed, indicating formation of VP_D_^+^ in P1 (up to approx. 82%, Fig. S26a†). At this point the system still contained approx. 2.3 eq. of unreacted DVP as well as the newly formed C3Ms. At 119 h SH (1.0 eq.) was added, which rapidly (within 2 h) and nearly exclusively reacted with the cationic VP_D_^+^ to produce neutral VP in P1 and the waste product DVP-SH (Fig. 5a–c). This can be concluded as only 3% of unreacted DVP was consumed during the formation of 23% DVP-SH. Similarly, 2 h after SH addition, both the scatter count and number average size had reduced to near starting levels (from 6.0 Mcps and 21 nm to 3.0 Mcps and 10 nm), confirming the expected C3M disassembly. Importantly, since there is still DVP in the system, this state of disassembly is not at equilibrium and spontaneous C3M re-assembly followed. After 312 h the number average size had returned to around 20 nm and the scatter count had reached 80% of the previous cycle peak. At this point DVP conversion had reached 50%, indicating a similar VP_D_^+^ species generation as in the first cycle. It could therefore be concluded that the system had returned to a near equilibrium C3M state. In an attempt to attain a second transient disassembly, a second SH (1.0 eq.) addition was made at 312 h. This resulted in further formation of DVP-SH (up to 53%) as well as reduced scatter count and number average size (from 4.8 Mcps and 20 nm to 2.8 Mcps and 10 nm). Re-assembly of C3Ms after this second disassembled state, was however, not observed despite the system theoretically containing enough DVP to allow for further VP_D_^+^ generation. Discrete DLS measurements recorded up to *t* = 1000 h indicated only a slight increase in scatter count (3.3 Mcps).

After successfully attaining a non-equilibrium C3M disassembly with DVP, we investigated if similar behaviour could be accessed with ME. Similarly, C3Ms were initially formed by addition of excess ME (3.0 eq.) to a solution of P1 and PSS (both 4 mM). The higher reactivity of ME resulted in rapid increases in scatter count and number average size, which plateaued after only 1 h, indicating stable assembly of micelles. At 18 h 30% of ME was consumed and SH (1.0 eq.) was added, resulting in a short-lived non-equilibrium C3M disassembly. This was demonstrated by reductions in both scatter count and number average size, with a minimum in these parameters 10 minutes after SH addition (from 6.0 Mcps and 21 nm to 3.7 Mcps and 13 nm). This was then followed by spontaneous C3M re-assembly and within 2 h both parameters had returned to near starting values. Interestingly, unlike the DVP based experiment a second transient disassembled state was attained after a second addition of SH (1.0 eq.) at 42 h. The time for C3M re-assembly was longer in this case (16 h), likely related to the reduced excess of remaining ME. TEM images from a sample taken at 65 h confirmed the reformation of spherical micelle structures after the second SH addition of 17.5 ± 2.7 nm (Fig. S29b†). Lastly, a final SH (1.0 eq.) addition resulted in sustained C3M disassembly (scatter count remained at 3.2 Mcps), with complete conversion of the ME to its waste products (ME-SH and ME-2SH) observed by ^1^H NMR (Fig. 5d–f). It is of note that despite the higher reactivity of ME, little direct reaction was observed between ME and SH. This experiment also demonstrated that increased ME concentration can reduce micelle assembly times, for example from approximately 6 h in the previous sequential addition experiment (Fig. 4c, 4 mM), to 1 h (Fig. 5e, 12 mM). Thus, in addition to increasing AA reactivity and reaction temperature, increasing AA concentration can also be used to accelerate C3M assembly.

Overall, these experiments demonstrate how pairing the relatively rapid kinetics of P1 deionization by SH with a slower, but in stoichiometric excess ionization of P1 by an AA, allows access to a transient non-equilibrium disassembly.

### Fuel-driven transient C3M assembly

**Fig. 6:**
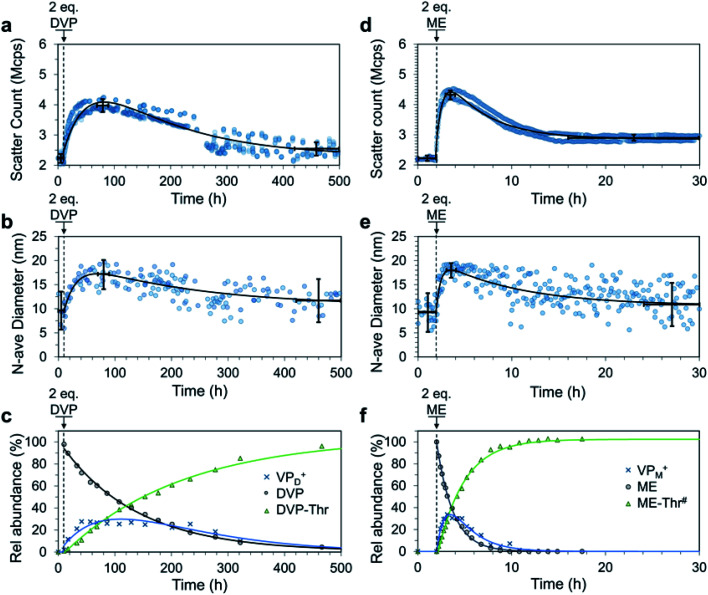
Fuel-driven transient C3M assembly by addition of 2.0 eq. fuel (DVP or ME) to a solution of P1 (4 mM), PSS (4 mM) and Thr (32 mM for (a–c) and 20 mM for (d–f)). Solutions were maintained at pH 7.4 by 250 mM PB buffer. DLS measurements run in triplicate on independent samples quantitating the scatter count (a and d) and number average diameter (b and e). Two-exponential curves (black line) were fit to the data. Average values (centre of cross) and standard deviation (vertical bars) before fuel addition, at peak micelle assembly and at the end were analysed assuming ∼ constant values over the time span shown by the horizontal bars. The process was also monitored by ^1^H NMR (c and f), where only fuel and waste signals were quantitated since PSS was included in the ^1^H NMR experiment. In the case of the ME fuelled experiment multiple waste products were formed, the total of which is described by ME-Thr^#^. Values for VP_D_^+^ and VP_M_^+^ are only an estimate, obtained by subtracting the amount of waste produced from the amount of fuel consumed. The values are expressed as a % conversion of VP in P1. Single and two-exponential curves are drawn to guide the eye. The break in DLS data in figures a and b between 240 and 280 h are due to a required filtration of all samples to remove an unidentified biological growth that began to accumulate in each sample. Filtration was through a 0.45 μm filter and therefore should not significantly remove the ∼20 nm micelles, for further discussion see Fig. S32 and S33.† Additional DLS, ^1^H NMR and TEM data can be found in Fig. S30–S36.†

We next envisaged that non-equilibrium assembled structures could be attained by reducing the reactivity of the deactivating nucleophile species such that the ionization reaction is faster than the de-ionization. In this way, a system could be constructed with an AA acting as a chemical fuel, allowing for an initial accumulation of the ionized VP units causing C3M assembly. If the nucleophile is present in excess it should eventually consume all ionized VP species after the fuel is depleted, ultimately leading to C3M disassembly (Fig. 1d). To achieve this behaviour, threonine (Thr), a relatively weak, primary amine nucleophile was selected based on our earlier work.^35^ After trialling various concentrations of P1, PSS, Thr and fuel, the following were found to be most optimal. First, we prepared pH 7.4 buffered solutions containing P1 (4 mM), PSS (4 mM) and Thr (32 mM) in triplicate. After 10 h to record baseline DLS data (2.2 ± 0.16 Mcps, 9.6 ± 4.1 nm), DVP fuel (2.0 eq., 8 mM) was added to trigger C3M assembly, leading to an increase in scatter count and number average size (Fig. 6a and b). These values peaked at 70–90 h (4.0 ± 0.21 Mcps, 17 ± 3.3 nm), after which a steady decrease in both parameters was observed. Between 420 and 500 h the scatter count and number average size had returned to near starting levels (2.5 ± 0.22 Mcps, 12 ± 4.4 nm), indicating transient assembly of C3Ms in solution. Note that scatter count and number average size values reported are an average ± standard deviation across triplicate measurements for the time period specified (where they are assumed to be ∼ constant).

In a similar case, ME fuel (2.0 eq., 8 mM) was added to pH 7.4 buffered solutions containing P1 (4 mM), PSS (4 mM) and Thr (20 mM). In this case, we recorded baseline DLS data for the first 2 h (2.3 ± 0.16 Mcps, 9.4 ± 4.1 nm), after which fuel addition caused rapid C3M assembly (Fig. 6d and e). Scatter count (4.3 ± 0.15 Mcps) and number average size (18 ± 1.6 nm) peak values were recorded between 3 and 4 h (1–2 h after ME addition). A spontaneous C3M disassembly followed, with scatter count reduced to 2.9 ± 0.1 Mcps at 16 h where it remained for the rest of the experiment. The number average size underwent a slower reduction to reach 11 ± 4.7 nm between 24 and 30 h.

In a separate experiment studied by DLS we achieved two fuel-driven C3M assembly cycles by ME additions (1.5 eq., 6 mM, twice) to a cuvette containing P1 (4 mM), PSS (4 mM) and Thr (24 mM) (Fig. S38†). Interestingly the rate of C3M assembly was reduced during the second cycle, perhaps due to accumulation of wastes in the system.^9^

It is of note that for both DVP and ME fuelled experiments, the peak number average size and scatter count were lower than those attained for signal-induced C3M assembly. This suggests a reduced or incomplete association of the polymers into micelles. Indeed, ^1^H NMR measurements indicated peak conversion to ionized pyridine species of only 27% (VP_D_^+^, Fig. 6c) and 34% (VP_M_^+^, Fig. 6f). This estimation was obtained by quantitating the excess consumption of fuel (DVP or ME) compared to waste(s) production. With ME as a fuel we observed multiple waste products, likely the result of ME-Thr further reacting as a nucleophile (secondary amine residue) with ME or reacting as a Michael acceptor with Thr (Fig. S34 and S35†). TEM images from samples taken at peak aggregation (100 h for DVP and 3.5 h for ME) were able to identify spherical micelle objects of 12.8 ± 4.7 nm and 16.2 ± 3.4 nm, respectively. These are slightly smaller than the diameters from corresponding signal-induced experiments and, unlike all previous measurements these were only able to be visualised effectively without uranyl acetate stain (Fig. S36 and S37†). With these considerations in mind, the results pleasingly demonstrate transient (partial) assembly of polymers into C3Ms with fuel dependent lifetimes.

## Conclusions

In conclusion, we have demonstrated fuel-driven coacervation within complex coacervate core micelles (C3Ms) at pH 7.4 using readily synthesisable polyamine block copolymers. This was achieved using a chemical reaction network (CRN) which is able to reversibly quaternize tertiary polyamine units. The CRN is fuelled with electron deficient allyl acetates where the reverse deionization reaction is controlled by addition of thiol or amine nucleophiles. We show that with appropriate polyanions this process allows for repeated (dis)assembly of C3Ms by coacervate phase modulation. The timescales of C3M assembly were tuned from days to hours by varying the allyl acetate reactivity, while additions of 2-mercaptoethanol deformed the C3Ms in minutes. With excess fuel, C3M assembly was further accelerated and additions of 2-mercaptoethanol resulted in transient disassembly of the C3Ms. By optimising the deionization kinetics, we achieved transient fuel-driven C3M assembly using a starting excess of a weak nucleophile (threonine). We anticipate this fuel-driven and nucleophile controlled coacervation system will inspire developments into triggered nucleic acid delivery and non-equilibrium protocell research.

## Materials and methods

### Materials

*N*,*N*-Dimethylacrylamide (DMA, 99%) and 4-vinylpyridine (4VP, 95%) were obtained from Sigma Aldrich and passed through basic alumina prior to use to remove inhibitor. Diethyl(α-acetoxymethyl) vinylphosphonate (DVP)^20,42^ and methyl 2-(acetoxymethyl)acrylate (ME) were synthesised according to literature procedures.^43^

4-((((2-Carboxyethyl)thio)carbonothioyl)thio)-4-cyanopentanoic acid (CETCPA, 95%, ABCR), 3-(trimethylsilyl)-1-propanesulfonic acid sodium salt (DSS, 97%), 2-mercaptoethanol (SH, >99%), acetic anhydride (AA, >99%), dimethylaminopyridine (DMAP, >99%), l-threonine (Thr, >98%), Na_2_HPO_4_·H_2_O (99%), poly(sodium 4-styrenesulfonate) (PSS, MW 200 kDa), sodium 2-acrylamino-2-methylpropane sulfonate (AMPS, ABCR), tetraethylmethylene diphosphonate (TMP, 97%), triethylamine (Et_3_N, 98%), were all used as received and purchased from Sigma Aldrich or TCI Europe unless stated otherwise. D_2_O (99.96% D), CDCl_3_ (99.8% D), MeOD (99.8% D) were obtained from Eurisotop.

### Methods

#### NMR spectroscopy

^1^H NMR and ^13^C NMR spectra were recorded on an Agilent 400-MR DD2 spectrometer (400 and 100 MHz, respectively).

Polymerisation conversion (*ρ*) was calculated by monitoring reduction in the ^1^H NMR integrals of the monomer unsaturated protons (∫*M*: 5.6–6.7 ppm for DMA, 5.5–6.7 ppm for 4VP) and aromatic protons in case of 4VP (7.5 ppm) relative to the internal standard DSS (0 ppm). In the case of a copolymerisation with both DMA and 4VP the conversion of both monomers was calculated (eqn (1)).1
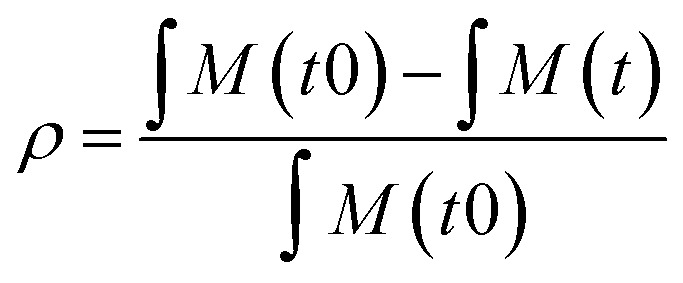


For a polymerisation containing z monomers, *M*_*n*,conv_ was calculated according to eqn (2). Here [*M*_*x*_]_0_ is the initial concentration of monomer *x*, [CTA]_0_ is the initial chain transfer agent (CTA) concentration and *M*_M*x*_ and *M*_CTA_ are the monomer *x* and CTA molecular weights, respectively.2
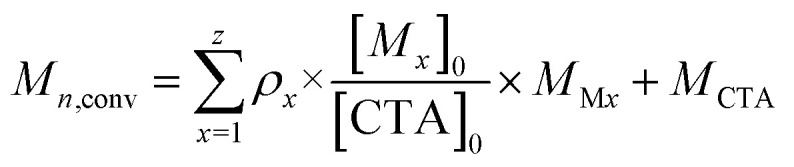


#### Gel permeation chromatography

The average molecular weight and dispersity (*Đ*) of the synthesized polymers was measured through either a Shimadzu GPC with DMF LiBr (25 mM) as eluent or a Shimadzu GPC with aqueous pH 8.0 buffer as eluent. The DMF system was equipped with a Shimadzu CTO-20AC Column oven, a Shimadzu RID-10A refractive index detector, a Shimadzu SPD-20A UV-vis detector, PLgel guard column (MIXED, 5 μm), 50 mm *×* 7.5 mm, and 1× Agilent PLGel (MIXED-C, 5 μm), 300 mm × 7.5 mm, providing an effective molar mass range of 200 to 2 × 10^6^ g mol^−1^. DMF LiBr (25 mM) was used as an eluent with a flow rate of 1.0 mL min^−1^ at 50 °C. The GPC columns were calibrated with low dispersity PMMA standards (Sigma Aldrich) ranging from 800 to 2.2 × 10^6^ g mol^−1^, and molar masses are reported as PMMA equivalents. The aqueous system was equipped with a Shimadzu CTO-20AC column oven, a Shimadzu RID-20A refractive index detector, PL aquagel-OH guard column (8 μm), 50 × 7.5 mm, and 2× Agilent PL-AquaGel-OH columns (Mixed H, 8 μm), each 300 mm × 7.5 mm^2^, providing an effective molar mass range of 100 to 10^7^ g mol^−1^. Aqueous buffer was prepared containing 80% (0.20 M NaNO_3_, 0.01 M NaH_2_PO_4_ in DI water) and 20% methanol adjusted to pH 8 and filtered through a 0.45 μm PTFE filter. The filtered aqueous buffer was used as an eluent with a flow rate of 1.0 mL min^−1^ at 40 °C. The GPC columns were calibrated with low dispersity PEO standards (Sigma Aldrich) ranging from 238 to 969 000 g mol^−1^, and molar masses are reported as PEO equivalents. A 3rd-order polynomial was used to fit the log *M*_p_*vs.* time calibration curve for both systems, which was near linear across the molar mass ranges.

#### Dynamic light scattering

Measurements were carried out using a Malvern Zetasizer Nano ZS employing a 633 nm laser at a back-scattering angle of 173°. The samples were measured in BRAND semi-micro PMMA cuvettes (10 mm path length) sealed with parafilm to prevent solvent loss for over time experiments. Scatter count values reported are all normalised based on measured scatter count and attenuator. The temperature inside the cell was set at 25 °C for all experiments except for an elevated temperature experiment with DVP which was at 37.5 °C.

#### Transmission election microscopy

TEM measurements were performed on a JEOL JEM-1400 plus TEM operated at 120 kV. Samples were prepared by placing 3 μL of the solution onto carbon coated copper grids and incubating for 1 minute followed by blotting the excess solution onto filter paper. The samples were then washed (2×) with of milli-q water, followed by removing the excess onto filter paper. The samples were then stained with uranyl acetate solution (2 wt%, 3 μL) for 30 seconds before removing the excess onto filter paper.

#### Polymer synthesis

Polymers were synthesised using photo-RAFT polymerisation with a LED reactor constructed from a 5 meter strip of 300 RGB 5050 SMD LEDs. These were procured from Ryslux (ebay) with *λ*_ma*x*_ of the blue lights measured to be 441 nm (Ocean Optics USB 4000 fibre coupled spectrometer). These LEDs were wound around a glass beaker of diameter 10 cm.

The water-soluble block (pDMA_261_) was first synthesised as follows. CETCPA (82.2 mg, 0.27 mmol), DMA (7.93 g, 80 mmol), DSS (21.3 mg, 0.10 mmol) and DI water (11.7 mL) were combined in a glass tube sealed with rubber septum. The reaction mixture was deoxygenated by bubbling with argon for 30 minutes and placed into the LED reactor (441 nm). The reaction was quenched after 2 hours irradiation (87% conversion by ^1^H NMR spectroscopy, *M*_*n*,conv_ = 26.2 kDa) by removing the glass tube from the light source and opening to air. The polymer was then purified by dialysis using spectra/por cellulose ester tubing (MWCO 500–1000 Da), followed by freeze drying to obtain a light-yellow powder.

The polyamine block-copolymer (P1) was then synthesised by chain-extending pDMA_261_ as follows. pDMA_261_ (536 mg, 0.020 mmol), 4VP (107 mg, 1.0 mmol), DMA (202 mg, 2.0 mmol), DSS (4.3 mg, 0.02 mmol) and MeOD (1.43 mL) were combined and deoxygenated by bubbling with argon for 15. The solution was then injected into a degassed NMR tube sealed with a rubber septum and placed into the LED reactor (441 nm). The reaction was quenched after 42 h irradiation (84% conversion of 4VP and 35% conversion of DMA by ^1^H NMR spectroscopy) by removing the glass tube from the light source and opening to air. The polymer was then diluted with ethanol and twice precipitated into diethyl ether (125 mL). The precipitate was then dissolved in DI water and dialysed using Spectra/por cellulose ester tubing (MWCO 500–1000 Da) followed by freeze drying to obtain a white powder. Both pDMA_261_ and P1 were analysed by GPC (DMF LiBr 25 mM), with low dispersities (*Đ* ≤ 1.20) recorded for both polymers. Successful chain extension was supported by a shift of a single peak from 28.5 kDa (pDMA_261_) to 32.9 kDa (P1). See ESI† for full GPC and ^1^H NMR characterisation data.

P2 and P3 were synthesised similarly using photo-RAFT polymerisation of AMPS, with full details provided in the ESI.†

#### Complex coacervate core micelle assembly (general)

All samples were prepared in either a 100 mM or 250 mM phosphate (PB) pH 7.4 buffer. Experiments using threonine (Thr) as the nucleophile used the higher 250 mM PB concentration while all other experiments used 100 mM PB. When NMR analysis was required, approximately 25% D_2_O was included in the buffer. Values reported are the average from the integration of multiple peaks, as shown for each experiment in ESI.† The concentrations given for the polyamines refer to the concentration of amine functionality in solution, while for polyanions it refers to the concentration of anionic functionality. All C3Ms were prepared at [amine functionality] : [anionic functionality] = 1 : 1.

For all time resolved experiments the polyanion used was PSS. Micelle assembly was triggered by additions of an allyl acetate fuel (DVP or ME), causing formation of cationic polyamine segments in P1 (VP_D_^+^ and VP_M_^+^). This ionization allowed for P1 to complex with the surrounding PSS into C3Ms. The nucleophile signal for micelle disassembly (deionizing VP_D_^+^ or VP_M_^+^ to VP in P1) was either mercaptoethanol (SH) or threonine (Thr). The thiol SH was always added from a freshly prepared stock solution (typically ∼0.4 M) in 100 mM PB pH 7.4 buffer, 10–20 μL additions). Similarly, the amine Thr was added from a stock solution in 100 mM PB pH 7.4 buffer. Additions of DVP and ME that were less than ∼4 mg were made from stock solutions (typically 40 mg mL^−1^ in PB buffer), which were stored in the fridge for a maximum of 1 week. Additions of DVP, ME, SH and Thr are all reported as equivalents with respect to the amount of P1 (pyridine functionality). Where applicable, 100 μL samples were taken for TEM and stored in the fridge before analysis. TEM samples of dissipative (non-equilibrium) states were fixed on the TEM grid within 1 hour of sampling.

To allow for analysis of the transition from pyridine to cationic pyridine protons by ^1^H NMR, the initial signal-induced micelle (dis)assembly experiments (20 mM) were conducted without the polyanion PSS. This prevents *in situ* formation of C3Ms during ^1^H NMR analysis, which can conceal signals in the core of the micelle. After confirming the aromatic proton shifts, later ^1^H NMR experiments (from transient C3M assembly and disassembly experiments) were conducted with PSS included to allow for any alterations to reactivity caused by micelle core localization of some reactants.

For fuel-driven transient C3M assembly experiments DLS data was recorded in triplicate (three independent samples). Values are reported as the mean from the 3 measurements, with selected error bars representing SD at that time range. The data was fit with a two exponential function (see eqn (3)), as can be crudely derived for the system if both forward and back reactions are assumed to be pseudo first order reactions.

3 *y* = *ae*^−*bx*^ + *ce*^−*dx*^ + *f*

## Data availability

Additional experimental details provided in ESI.† Raw experimental data including TEM images (.tif), NMR spectra (.fid) and DLS data (.xlsx) have been uploaded to the 4TU repository. https://doi.org/10.4121/1950036.

## Author contributions

R. L. investigation, writing – original draft. B. K. investigation. M. M. resources. R. E. conceptualization, funding acquisition, writing – review & editing.

## Conflicts of interest

There are no conflicts to declare.

## Supplementary Material

SC-013-D2SC00805J-s001
