# Supplementary material for: Fuel-driven macromolecular coacervation in complex coacervate core micelles
Source: Chem Sci. 2022 Mar 31;13(16):4533–44. doi: 10.1039/d2sc00805j (PMC9019912; doi:10.1039/d2sc00805j)
Supplement: SC-013-D2SC00805J-s001 [file SC-013-D2SC00805J-s001.pdf]

Electronic Supplementary Material (ESI) for Chemical Science.
This journal is © The Royal Society of Chemistry 2022

Supporting information

Fuel-Driven Macromolecular Coacervation in Complex Coacervate Core Micelles

Reece W. Lewis, Benjamin Klemm, Mariano Macchione, Rienk Eelkema*

Department of Chemical Engineering, Delft University of Technology, Van der Maasweg 9, 2629 HZ Delft, The Netherlands.

* Correspondence to: R.Eelkema@tudelft.nl

Table of Contents

Polymer synthesis 2

Polycation block copolymer (pDMA$_{261}$ and P1) synthesis 2

Polyanion (P2 and P3) synthesis 2

Allyl acetate synthesis 8

Diethyl(α-acetoxymethyl) vinylphosphonate (DVP) 8

Methyl 2-(acetoxymethyl)acrylate (ME) 10

C3M morphology study 11

Signal-induced C3M (dis)assembly 17

Transient C3M disassembly 28

Fuel-driven transient C3M assembly 34

References 44

Polymer synthesis

Polycation block copolymer (pDMA$_{261}$ and P1) synthesis

The synthetic procedure is provided in the main text, with a summary of conditions provided in **Table S1.** GPC data is presented in **Table S3** and **Figure S1**, while ^{1}H NMR traces are shown in **Figures S2 – S3**.

Polyanion (P2 and P3) synthesis

The polyanions P2 and P3 were synthesised as follows, with synthesis and characterisation data summarised in **Table S2**.

P2: CETCPA (82 mg, 0.27 mmol), AMPS (1.83 g, 8.0 mmol), DMA (2.37 g, 23.9 mmol), DSS (29.7 mg, 0.14 mmol), dioxane (0.55 mL) and DI water (7.0 mL) were combined in a glass tube sealed with rubber septum. The reaction mixture was deoxygenated by bubbling with argon for 30 minutes and placed into the LED reactor (441 nm). The reaction was quenched after 6.5 hours irradiation (83% conversion of AMPS and 87% conversion of DMA by ^{1}H NMR spectroscopy, $M_{n,conv}$ = 13.5 kDa) by removing the glass tube from the light source and opening to air. The polymer was then purified by dialysis using Spectra/por cellulose ester tubing (MWCO 500 – 1000 Da) followed by freeze drying to obtain a yellow powder.

P3: CETCPA (203 mg, 0.66 mmol), AMPS (4.57 g, 20 mmol), DSS (14.7 mg, 0.067 mmol), dioxane (0.50 mL) and DI water (7.5 mL) were combined in a glass tube sealed with rubber septum. The reaction mixture was deoxygenated by bubbling with argon for 30 minutes and placed into the LED reactor (441 nm). The reaction was quenched after 33 hours irradiation (83% conversion by ^{1}H NMR spectroscopy, $M_{n,conv}$ = 6.0 kDa) by removing the glass tube from the light source and opening to air. The polymer was then purified by dialysis using Spectra/por cellulose ester tubing (MWCO 500 – 1000 Da), followed by freeze drying to obtain a yellow solid.

The polyanions were then all analysed by aqueous GPC (pH 8), with both polymers having low dispersities (Đ = 1.15) and molecular weights in agreement with those obtained from NMR conversion (**Table S3**). See **Figures S4 – S5** for ^{1}H NMR spectra of the final purified polymers.

Table S1. Polyamine block-copolymer synthesis and characterisation data

Polymer	CTA	$[CTA]_0$: $[DMA]_0$: $[4VP]_0$	Polym time (h)	NMR Conv (%)	Structure	$M_{n,conv}$ (kDa)
**$pDMA_{261}$**	CETCPA	1:300:0	2.0	87 (DMA)	$p(DMA_{261})$	26.2
**P1**	$pDMA_{261}$	1:100:50	42	84 (4VP)<br>35 (DMA)	p[($4VP_{36}$-*c*-DMA_{28})-*b*-(DMA_{261})]	32.8

Table S2. Polyanion synthesis and characterisation data

Polymer	CTA	$[CTA]_0$: $[DMA]_0$: $[AMPS]_0$	Polym time (h)	NMR Conv (%)	Structure	$M_{n,conv}$ (kDa)
**P2**	CETCPA	1:90:30	6.5	83 (AMPS)<br>87 (DMA)	p($AMPS_{25}$-*c*-DMA_{75})	13.5
**P3**	CETCPA	1:0:30	33	83 (AMPS)	p($AMPS_{23}$)	6.0

Table S3. Molecular weight characterisation data for RAFT synthesised polymers.

Polymer	$M_{n,conv}$ (kDa)	$M_{n,GPC}$ (kDa)	*Đ*	*GPC system*
**$pDMA_{261}$**	26.2	28.5	1.15	DMF
**P1**	32.8	32.9	1.20	DMF
**P2**	13.5	16.1	1.15	Aqueous
**P3**	6.0	8.4	1.15	Aqueous

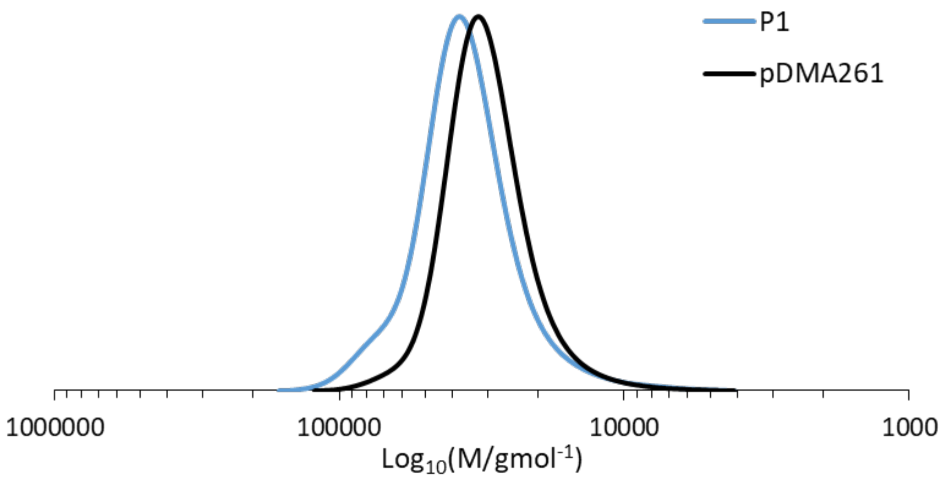

Figure S1. DMF LiBr (25 mM) GPC data showing chain extension from $pDMA_{261}$ to P1.

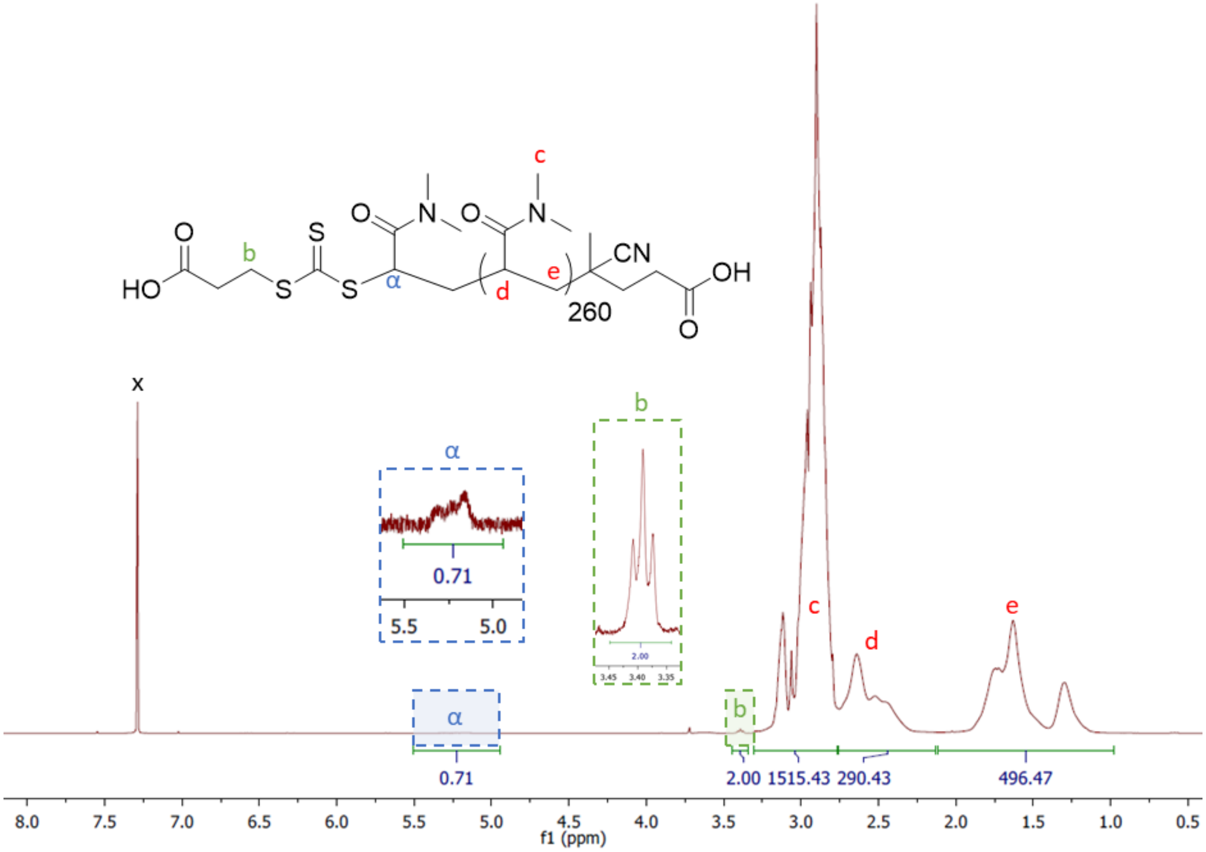

Figure S2. ^{1}H NMR ($CDCl_3$) of $pDMA_{261}$ demonstrating good agreement (±5%) between polymer structure determined by ^{1}H NMR conversion and ratio of end-group signals to polymer backbone. $\int c_{th} = 261 \cdot 6 = 1566$; $\int d_{th} = 261 = 261$; $\int e_{th} = 261 \cdot 2 = 522$.

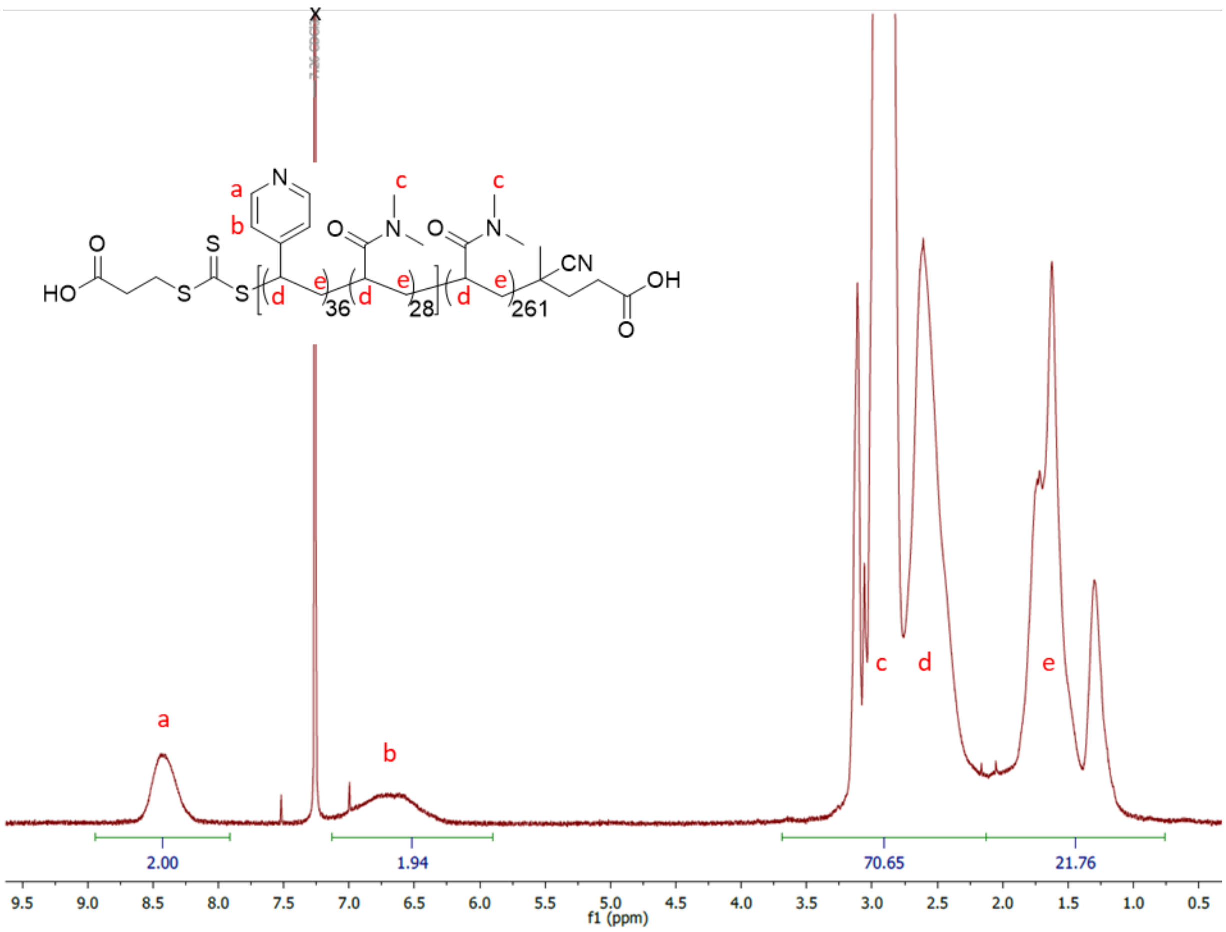

Figure S3. ^{1}H NMR ($CDCl_3$) of P1 demonstrating reasonable (±20%) agreement between polymer structure determined by ^{1}H NMR conversion and ratio of p4VP aromatic signals to polymer backbone. $\int c{+}d_{th} = [6 \cdot (261 + 28) + 1 \cdot (36 + 28 + 261)] / 36 = 57.2$; $\int e_{th} = (2) \cdot (261 + 36 + 28) / 36 = 18.1$.

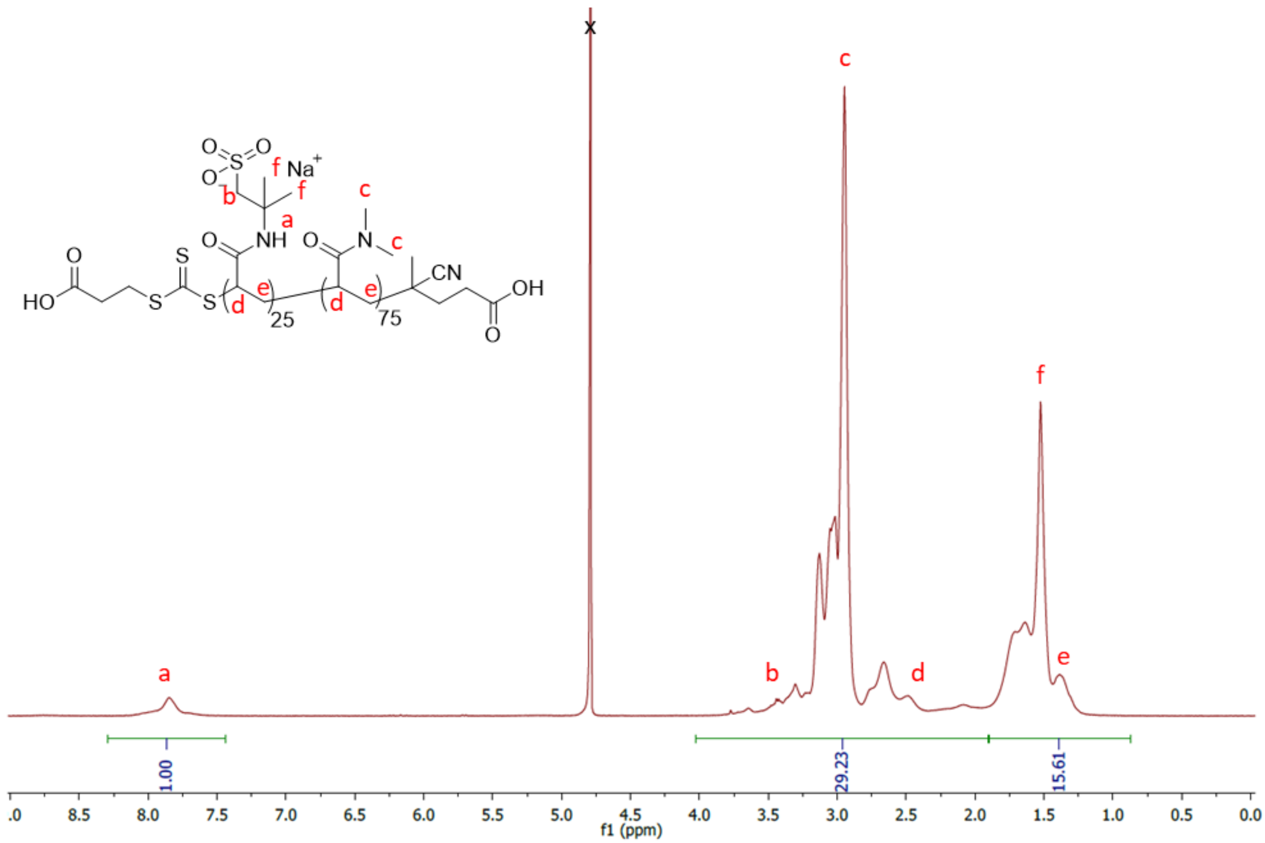

Figure S4. ^{1}H NMR (D_2O) of P2 demonstrating reasonable (±20%) agreement between polymer structure determined by ^{1}H NMR conversion and ratio of pAMPS N-H signal to polymer backbone. $\int b+c+d_{th} = [2 \cdot 25 + 6 \cdot 75 + 1 \cdot (25 + 75)] / 25 = 24$; $\int e+f_{th} = [6 \cdot 25 + 2 \cdot (25 + 75)] / 25 = 14$.

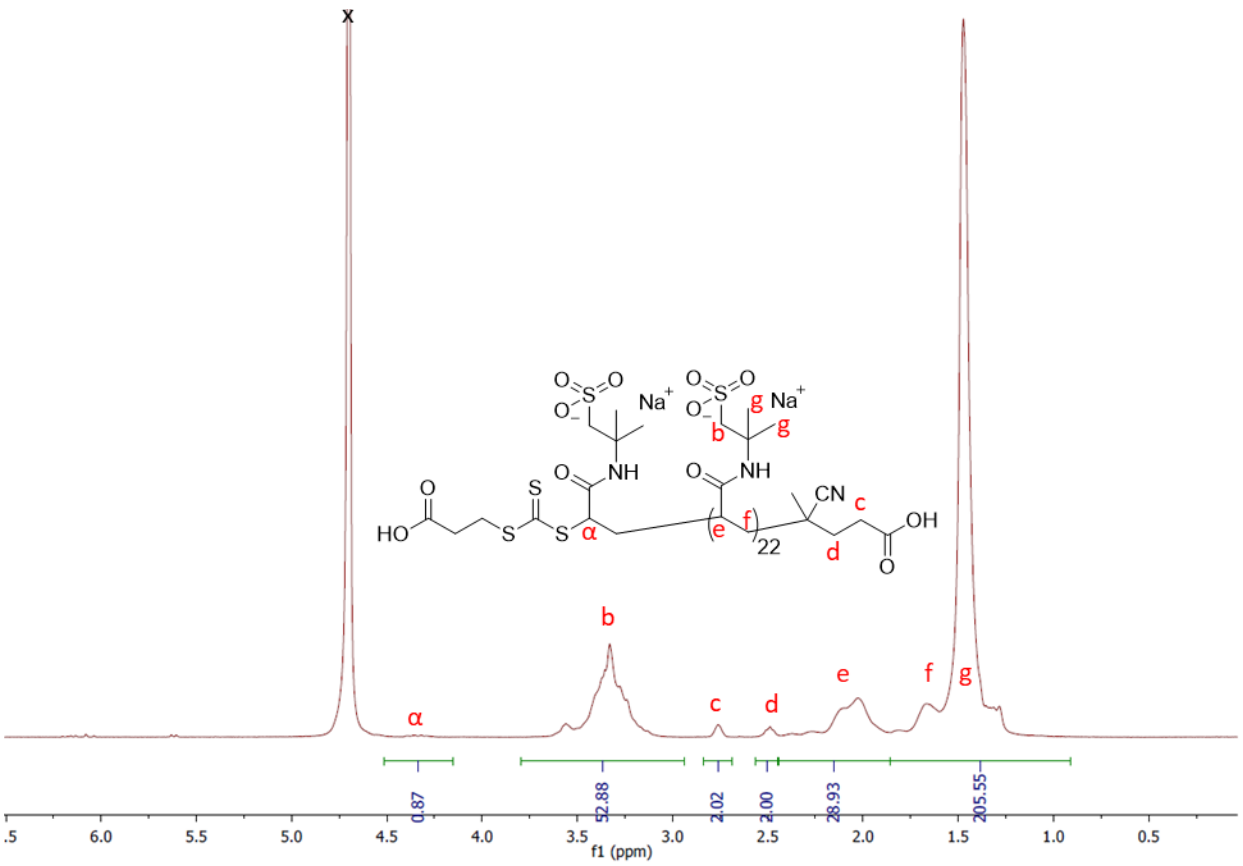

Figure S5. ¹H NMR (D_2O) of P3 demonstrating reasonable (±15%) agreement between polymer structure determined by ¹H NMR conversion and ratio of polymeric signals to end groups. $\int b_{th} = 2 \cdot 23 = 46$; $\int e_{th} = 1 \cdot 23 = 23$; $\int f+g_{th}\ (6 + 2) \cdot 23 = 184$.

Allyl acetate synthesis

Diethyl(α-acetoxymethyl) vinylphosphonate (DVP)

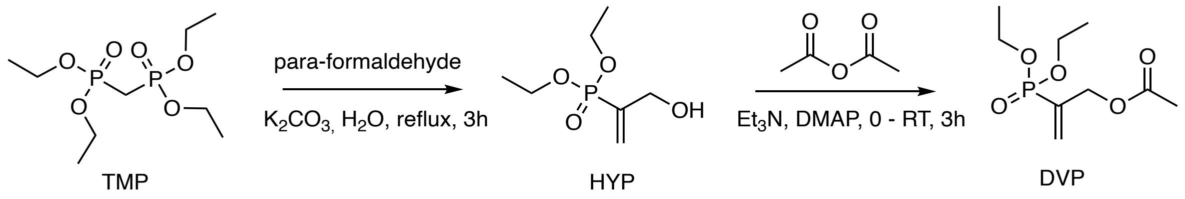

Scheme S1. Synthetic pathway for the preparation of DVP.

DVP is a known compound and was synthesized following reported procedures[1, 2] (Scheme S). Briefly, a mixture of tetraethyl methylene diphosphonate (TMP, 20.0 g, 69.4 mmol) in aqueous para-formaldehyde (30%, 50 mL) was heated under reflux. A solution of potassium carbonate (19.2 g, 138.8 mmol in 30 mL of H_2O) was slowly added over a period of 3 hours via a syringe pump. After cooling to room temperature, the reaction mixture was extracted with chloroform (5x 50 mL), washed with brine (2x 50 mL) and dried with Na_2SO_4. Then, the reaction mixture was concentrated under reduced pressure and the residue was subjected to vacuum distillation to afford diethyl (3-hydroxy-2-propenyl)phosphonate (HYP, 10.1 g, 75%) bp: 95 – 105 °C (0.001 torr) as a colourless oil.

HYP. ^{1}**H NMR** (400 MHz, $CDCl_3$) δ: 6.09 (d, J = 2.7 Hz, 1H), 6.01 (d, J = 20.0 Hz, 1H), 4.30 (d, J = 10.7 Hz, 2H), 4.20 – 4.00 (m, 4H), 2.30 (s, 1H), 1.33 (t, J = 7.0 Hz, 6H). ^{13}**C NMR** (100.5 MHz, $CDCl_3$) δ: 140.0, 138.3, 128.9 (d, J = 7.2 Hz), 62.6 (d, J = 16.0 Hz), 62.3, 16.4 (d, J = 6.4 Hz). ^{31}**P NMR** (161.9 MHz, $CDCl_3$) δ: 17.6 ppm. **MS** (ESI+) m/z: 195.06 (M+H) (expected m/z: 195.07).

To a cooled (0°C) solution of this phosphonate (6.0 g, 30.1 mmol), DMAP (0.227 g, 1.85 mmol) and Et_3N (6.46 mL, 46.35 mmol, 1.5 eq.) in dichloromethane (10 mL) was added dropwise a solution of acetic anhydride (3.21 mL, 34.0 mmol) in dichloromethane (100 mL) and the mixture was stirred at room temperature for 3 h. The reaction mixture was then washed with 15% Na_2CO_3 (2x 100 mL) until pH 9, with 5% HCl (pH 2) and brine before it was dried with Na_2SO_4. Then, the reaction mixture was concentrated under reduced pressure and the residue was purified by flash

chromatography (ethyl acetate/methanol, 95:5) to furnish diethyl(α-acetoxymethyl) vinylphosphonate as a colourless oil (DVP, 6.1 g, 84%).

DVP. ^{1}H NMR (400 MHz, $CDCl_3$) δ: 6.19 (dt, J = 22.4, 1.4 Hz, 1H), 6.01 (dt, J = 45.8, 1.6 Hz, 1H), 4.72 (dt, J = 8.9, 1.5 Hz, 2H), 4.23 – 3.93 (m, 4H), 2.09 (d, J = 1.3 Hz, 3H), 1.32 (t, J = 7.1, 1.3 Hz, 6H). **^{13}C NMR** (100.5 MHz, $CDCl_3$) δ: 170.3, 135.6, 133.8, 131.1 (d, J = 7.0 Hz), 63.1, 62.9, 62.3 (d, J = 5.7 Hz), 20.9, 16.4 (d, J = 6.3 Hz). **^{31}P NMR** (161.9 MHz, $CDCl_3$) δ: 15.9 ppm. **MS** (ESI+) m/z: 237.07 (M+H) (expected m/z: 237.08).

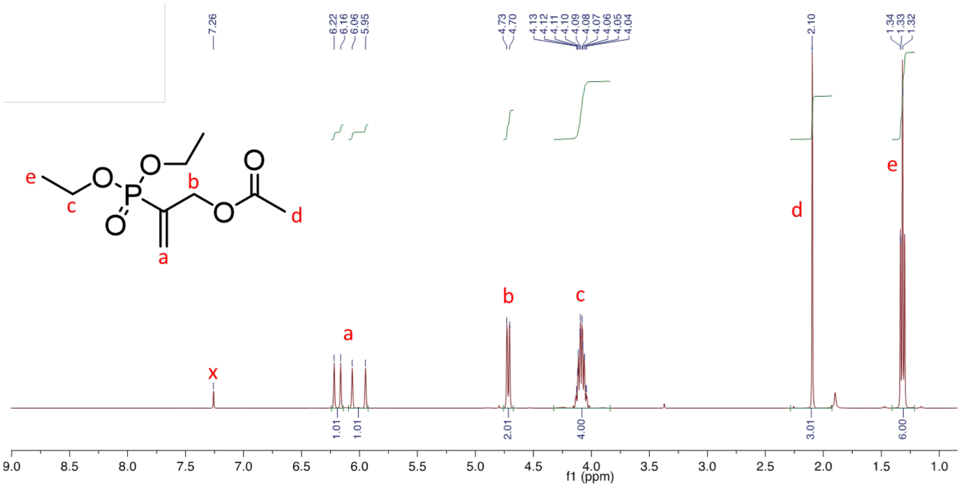

Figure S6: ^{1}H NMR ($CDCl_3$) of DVP.

Methyl 2-(acetoxymethyl)acrylate (ME)

ME was synthesised according to literature procedures with ^{1}H NMR in agreement with reported values.[3]

^{1}H NMR (400 MHz, $CDCl_3$): δ = 6.36 (m, 1H), 5.85 (m, 1H), 4.80 (s, 2H), 3.79 (s, 3H), 2.10 (s, 3H). **^{13}C NMR** (100 MHz, $CDCl_3$): δ = 170.89, 166.15, 135.66, 128.08, 62.93, 52.51, 21.33.

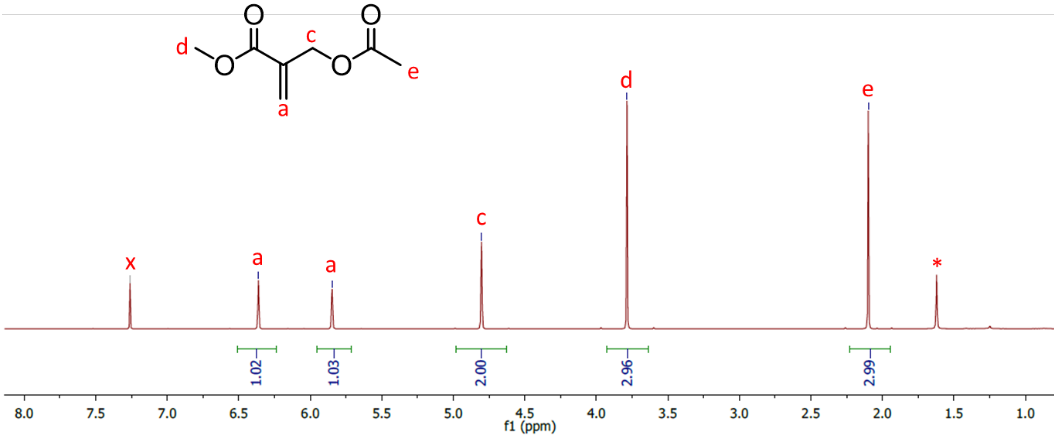

Figure S7. ^{1}H NMR ($CDCl_3$) of ME.

C3M morphology study

$P1_D^{23+}$ was prepared by addition of DVP (1.0 eq.) to P1 (20 mM) in 100 mM PB buffer (25% D_2O). Analysis by ^1H NMR spectroscopy demonstrated a noticeable shift in the aromatic pyridine signals, while a shift and broadening was observed for the DVP signals (see Figure S12). By quantitating these shifts, a 65% conversion to the cationic pyridine adduct in P1 (VP_D^+) was determined after 5 days reaction (**Figures S8** and **S10**). Based on 36 units of VP per P1 chain this equates to an average of 23 cationic units (VP_D^+) per chain. Similarly, $P1_M^{29+}$ was prepared by addition of ME (1.0 eq.) to P1 (20 mM) in 100 mM PB buffer (25% D_2O). This reaction reached 80% conversion in 5 hours, with 29 cationic units (VP_M^+) per chain (**Figures S9 -S10**).

The $P1_D^{23+}$ solution from above was filtered and diluted to 8 mM. The polycation solution was then combined with an equal volume of various polyanion stock solutions (P2, P3 and PSS; 8 mM) such that the final concentration of amine and anion functionality was 4 mM. The resultant species morphologies were then analysed by DLS and TEM (supporting data in **Figures S11 - S12**).

The TEM images proposed to contain micelles were analysed using Image J software by manually drawing spheres around 30 species per image to record an average diameter. Values reported in **Figure S11** are the average of at least two separate images analysed per sample, with standard deviation calculated as below (**Equation S3**).

$$\sigma^2{}_{meas} = \sigma^2{}_{across\ samples} + \sigma^2{}_{within\ samples} \qquad \textbf{Eq. S3}$$

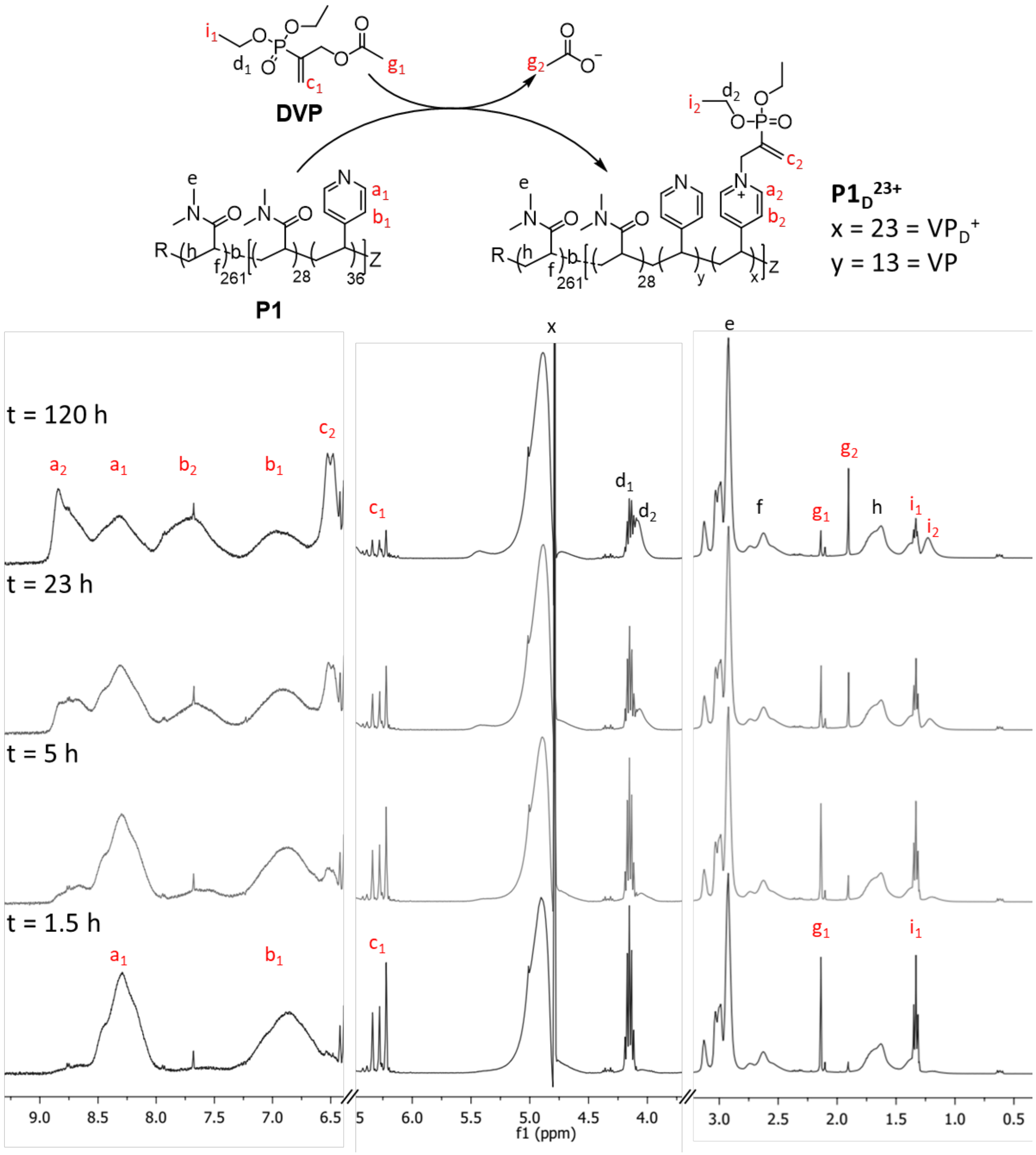

Figure S8. ^{1}H NMR (D_2O, pre-sat) of the pyrdine functionality in P1 reacting with DVP to form the ionized polyamine block copolmyer $P1_D^{23+}$ ([DVP] = [P1] = 20 mM). Process involves conversion of neutral pyridine units in P1 (VP) to quaternary charged adducts (VP_D^+). Peaks labelled in red were integrated to quantitate the extent of conversion at each time point measured. Note that spectra is cut into 3 rescaled segments for clarity. After 120 hours sample was mixed with various polyamines in the intial morphology study (Figure).

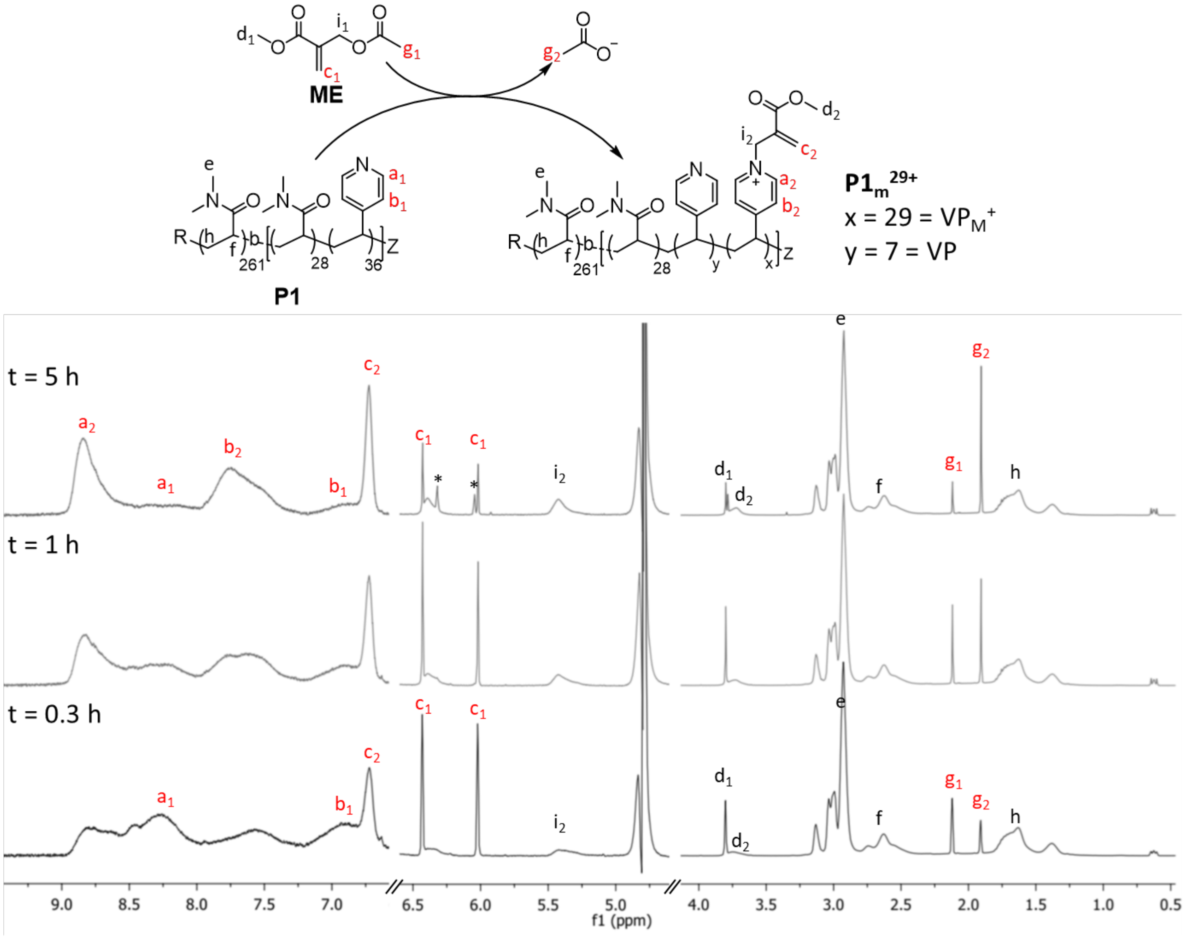

Figure S9. ^{1}H NMR (D_2O, pre-sat) of the pyrdine functionality in P1 reacting with ME to form the ionized polyamine block copolmyer $P1_M^{29+}$ ([ME] = [P1] = 20 mM). Process involves conversion of neutral pyridine units in P1 (VP) to quaternary charged adducts (VP_M^+). Peaks labelled in red were integrated to quantitate the extent of conversion at each time point measured. Note that spectra is cut into 3 rescaled segments for clarity. *Additional signals observed around 6.0 – 6.5 ppm at 5 h are believed to be related to phosphate nucleophilic substitution onto VP_M^+ (ME-PB, see **Figure S18**).

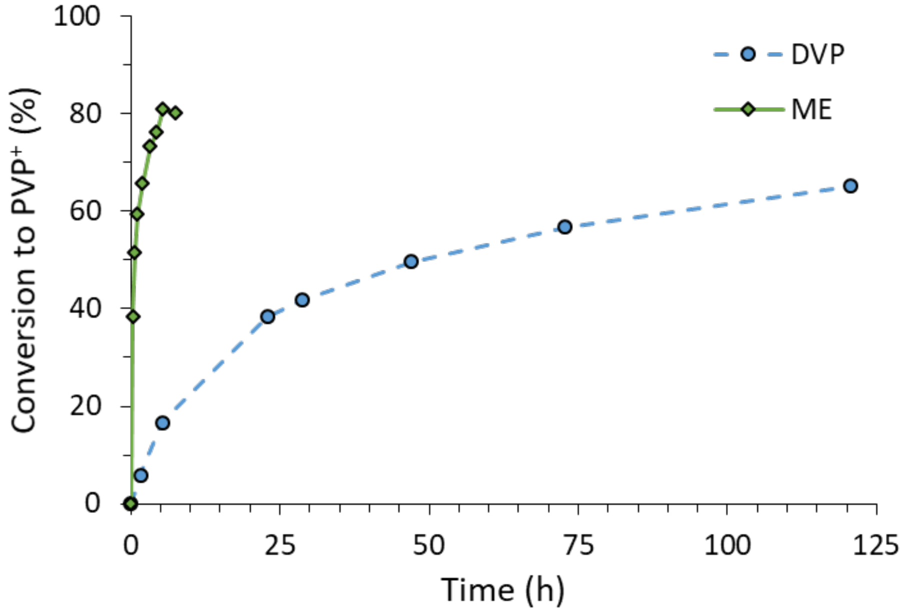

Figure S10. Conversion of neural tertiary amine functionality (VP) in P1 (20 mM) to charged quaternary amine (VP_D^+ / VP_M^+) by reaction with DVP / ME (1.0 eq.) in 100 mM PB buffer (approx. 25% D_2O) as monitored by ^{1}H NMR spectroscopy.

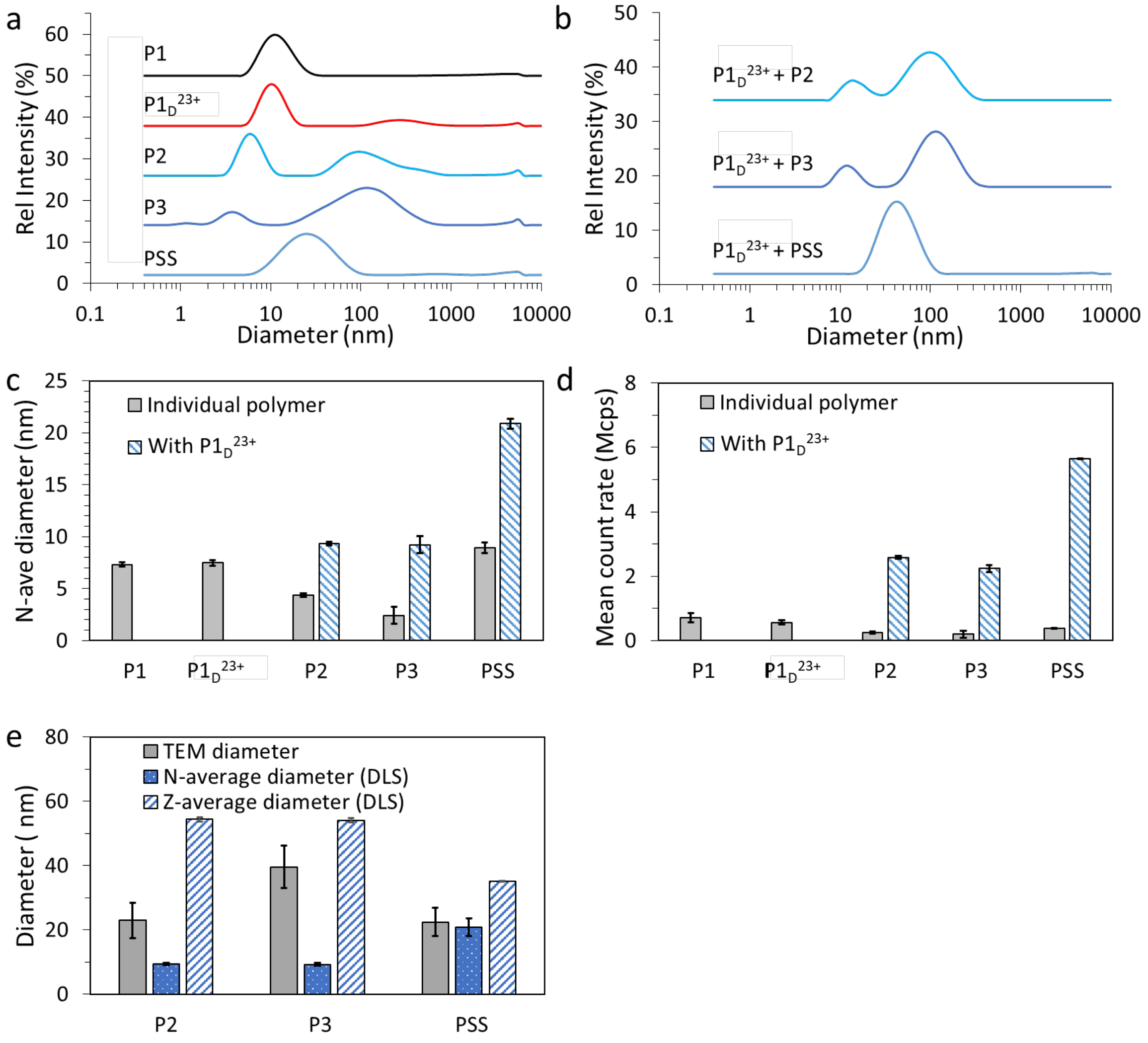

Figure S11. Additional characterisation data of P1, $P1_D^{23+}$ P2, P3, PSS and their corresponding C3Ms formed during morphology study in 100 mM PB pH 7.4 buffer, Figure 2. a) DLS intensity plots for each individual polymer ([amine/anionic functionality] = 8 mM). b) DLS intensity plots for C3Ms formed by combination of $P1_D^{23+}$ with each polyanion ([amine] = [anionic functionality] = 4 mM) demonstrating the effect of polyanion structure on C3M morphology. Of note is the bimodal peaks observed in P2 and P3, indicative of 2-dimensional (i.e. wormlike) species. c) Comparison of number average diameter (DLS) of polymers and their corresponding C3Ms formed with $P1_D^{23+}$. d) Comparison of normalised scatter count (DLS) of polymers and their corresponding C3Ms formed with $P1_D^{23+}$ ([amine] + [anionic functionality] = 8 mM). e) Comparison of TEM and DLS characterisation of C3M diameter. TEM diameter is the average of

30 species, measured manually using image J software. All DLS measurements are the mean of two independently prepared samples, each analysed 4 times. Error bars are ± SD.

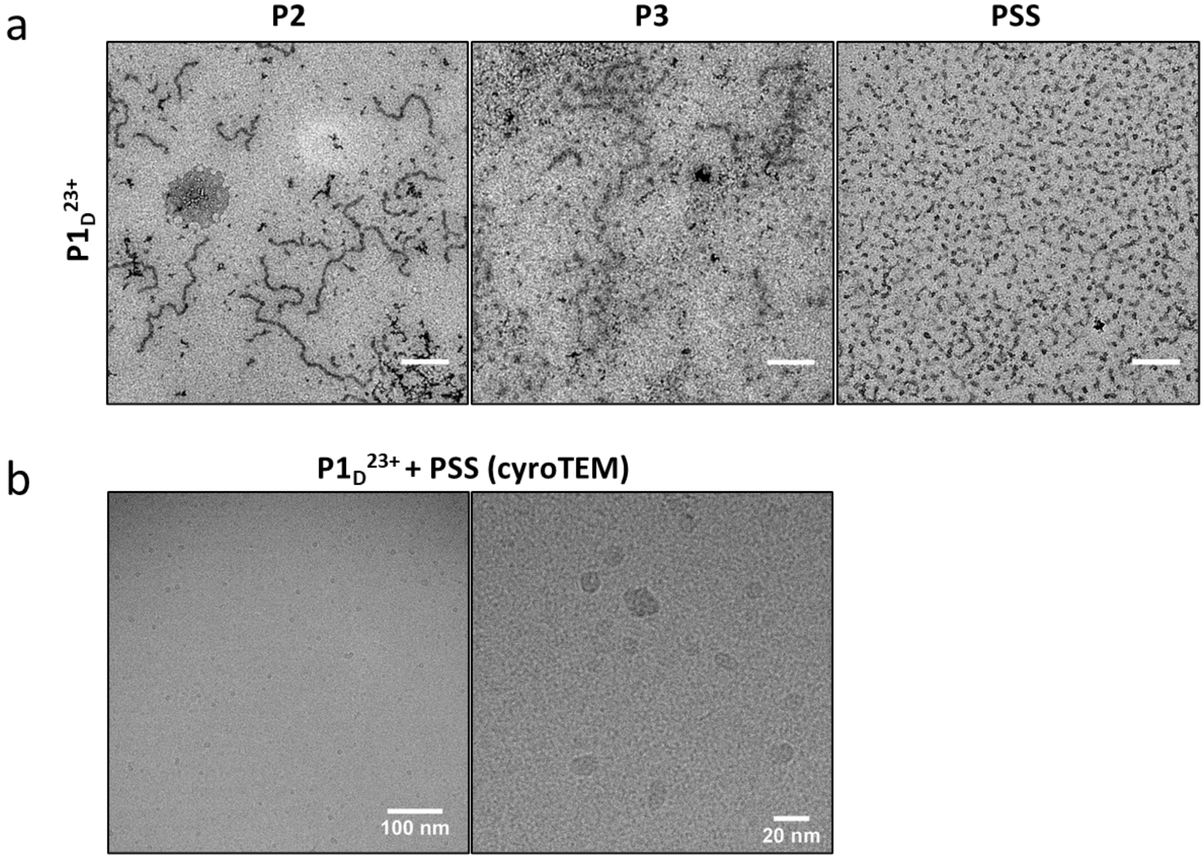

Figure S12. (a) Additional lower magnification TEM images from morphology study presented in Figure 2 (Scale bars 200 nm). (b) cyroTEM images of PSS + $P1D_{23}^{+}$. Images were analysed manually with ImageJ, yielding an average diameter of 10.6 nm (n = 80, SD = 2.5).

Cryo-EM (electron cryo-microscopy) images were obtained with a JEOL JEM3200-FSC, operated at 300kV with Gatan camera K2-Summit operated in counting mode with 10s exposure time, dose fractionated 0.2s aligned by SerialEM. Zero-Loss filtered with a 20eV slit. Samples were prepared with a Leica plunger EM GP, 21 °C, 98% RH, 8 seconds blotting time.

Signal-induced C3M (dis)assembly

Concentrated (20 mM) experiments

P1 (18.2 mg, 20.0 µmol amine), DVP (4.7 mg, 20 µmol), DSS (2 mg, 21 µmol) and 100 mM PB pH 7.4 buffer (1.0 mL, ~25% D_2O) were combined in an NMR tube, with the reaction monitored over time by ^1H NMR. Separately P1 (18.2 mg, 20.0 µmol amine), PSS (4.12 mg, 20.0 µmol sulfonate) and 100 mM PB pH 7.4 buffer (1.0 mL) buffer were combined and filtered (0.45 µm filter) followed by addition of DVP (4.7 mg, 20 µmol), then analysed by DLS over time. Sequential additions of SH and DVP (1.0 eq.) were then made to both the NMR and DLS samples. For the experiment with ME, the same procedure was followed with DVP replaced with ME (3.2 mg, 20 µmol). For select ^1H NMR spectra and additional DLS data from these experiments see **Figures S13 – S14** (DVP) and **Figures S18 – S21** (ME).

Dilute (4 mM) experiments

P1 (14.6 mg, 16.0 µmol amine), PSS (3.3 mg, 16.0 µmol sulfonate) and 100 mM PB pH 7.4 buffer (4.0 mL) were combined and filtered (0.45 µm filter). A 1.0 mL sample was taken as reference, then DVP (2.8 mg, 12 µmol) was added to the remaining 3.0 mL polymer solution and monitored by DLS over time. Sequential additions of SH and DVP (1.0 eq.) were then made. For experiments with ME as the allyl acetate, the same procedure was followed with DVP replaced with ME (2.5 mg, 16 µmol). For the DVP experiment conducted at 37.5°C (all other experiments at 25°C), heating was achieved in the DLS cell or by submerging the cuvette in a 38°C water bath. DLS data from the initial 25°C (room temperature) experiment with DVP is presented in **Figure S15**. Additional DLS and TEM data for the dilute (4 mM) experiments is presented in **Figures S16 – S17** (DVP) and **Figures S22 – S24** (ME), including a repeat of the ME experiment demonstrating great reproducibility.

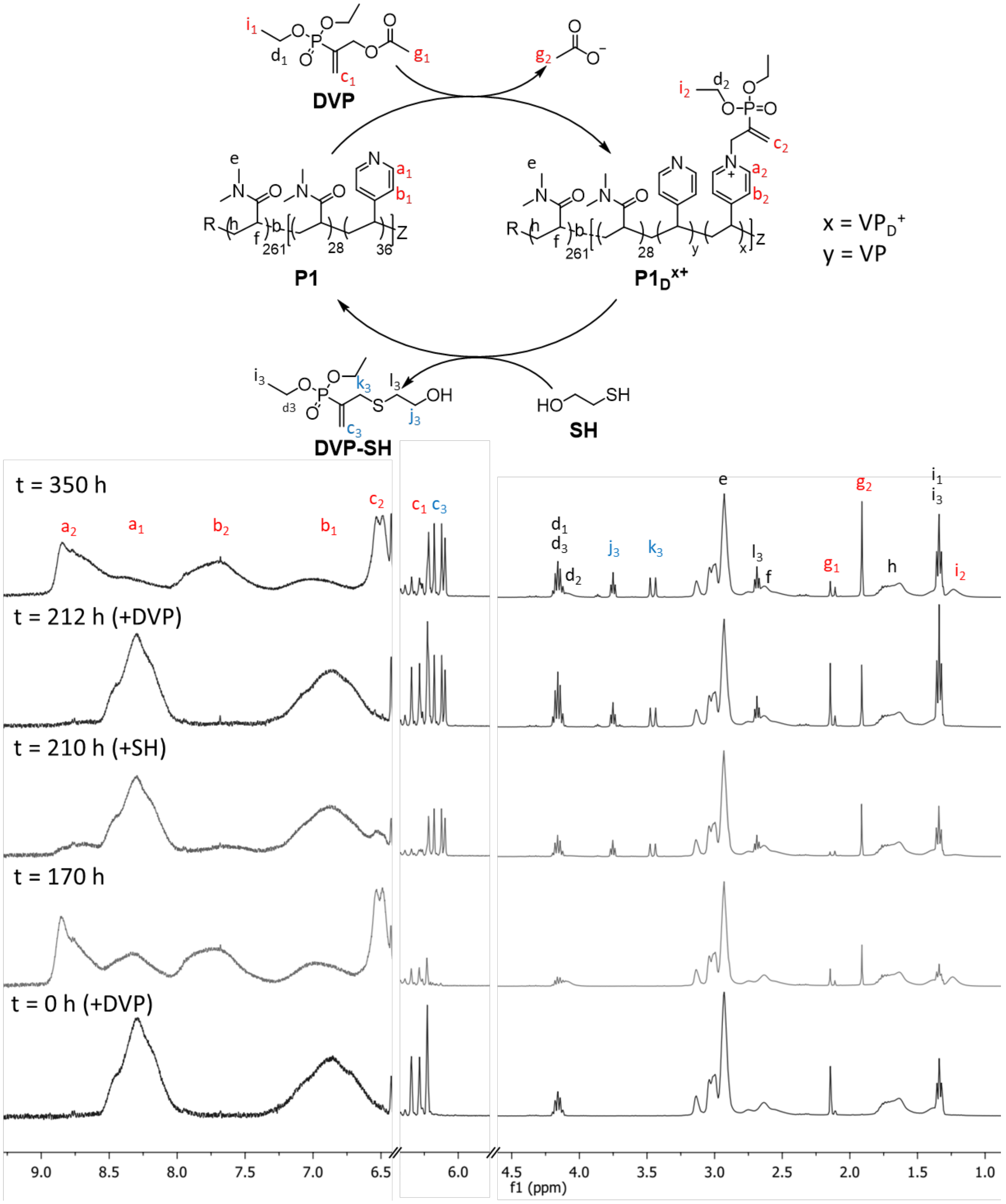

Figure S13. Example ^{1}H NMR (D_2O, pre-sat) spectra from DVP and thiol (SH) signal induced P1 (de)ionization experiment (See Figure 3, [P1] = 20 mM; DVP and SH additions at 1.0 eq). Note in the scheme x = 0 at t = 0. Peaks labelled in red and blue were integrated to quantitative the extent

of conversion to cationic pyridine adduct (VP_D^+) in P1 and waste (DVP-SH), respectively. Note that spectra is cut into 3 rescaled segments for clarity. Polyanion (PSS) was exlcuded from this experiment to avoid supression of signals in micelles.

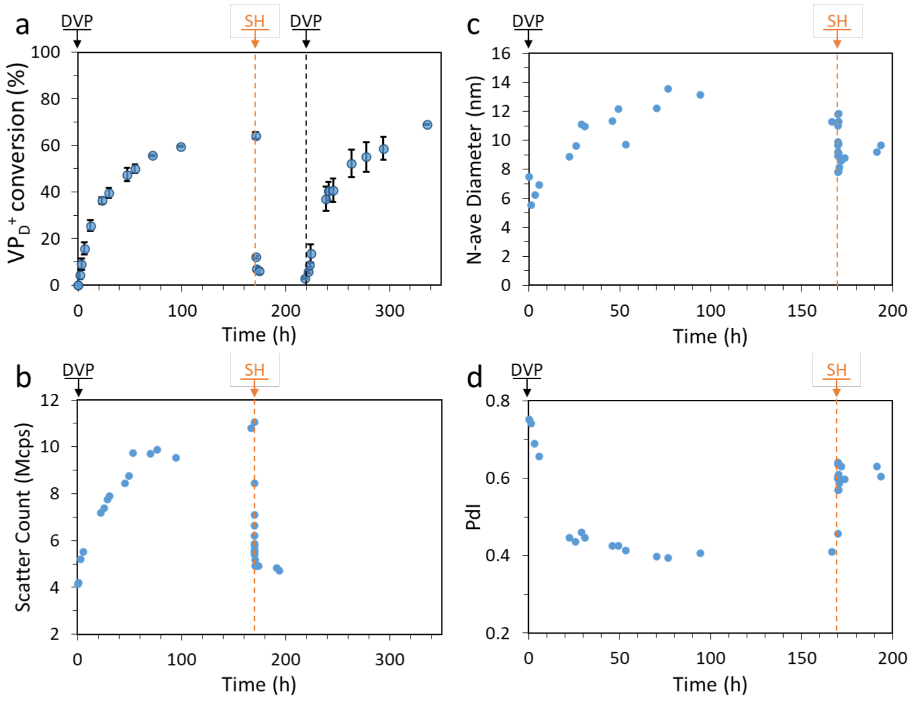

Figure S14. Additional DLS and NMR data for DVP and thiol (SH) signal induced C3M assembly and disassembly experiment (See Figure 3, [P1] = 20 mM; DVP and SH additions at 1.0 eq). For all except a) (NMR experiment) [PSS] = 20 mM. The conversion values reported in a) are as shown in Figure 3a and are an average from integration of shifting aromatic peaks, acetate CH_3 and DVP CH_3 with error bars ± SD of these different measurements. The less clear trends observed in N-ave diameter are attributed to issues arising from high polymer concentration (possibly multiple scattering).

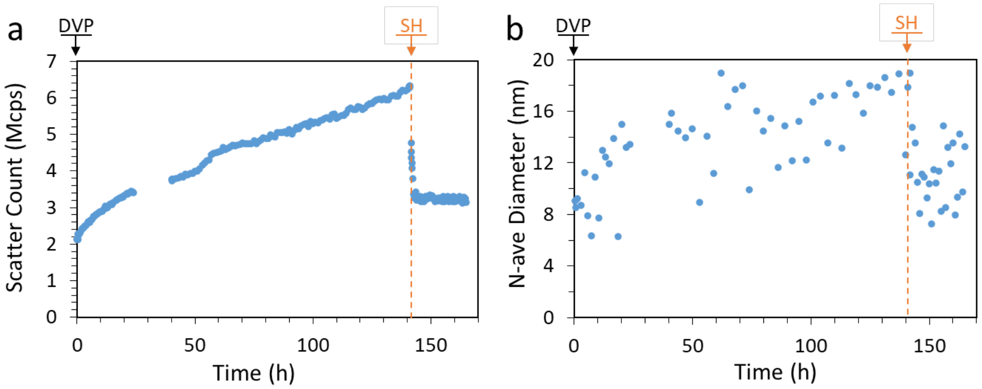

Figure S15. Full DLS data from DVP signal induced C3M assembly at 25°C (See Figure 3, [P1] = [PSS] = 4 mM; DVP and SH additions at 1.0 eq).

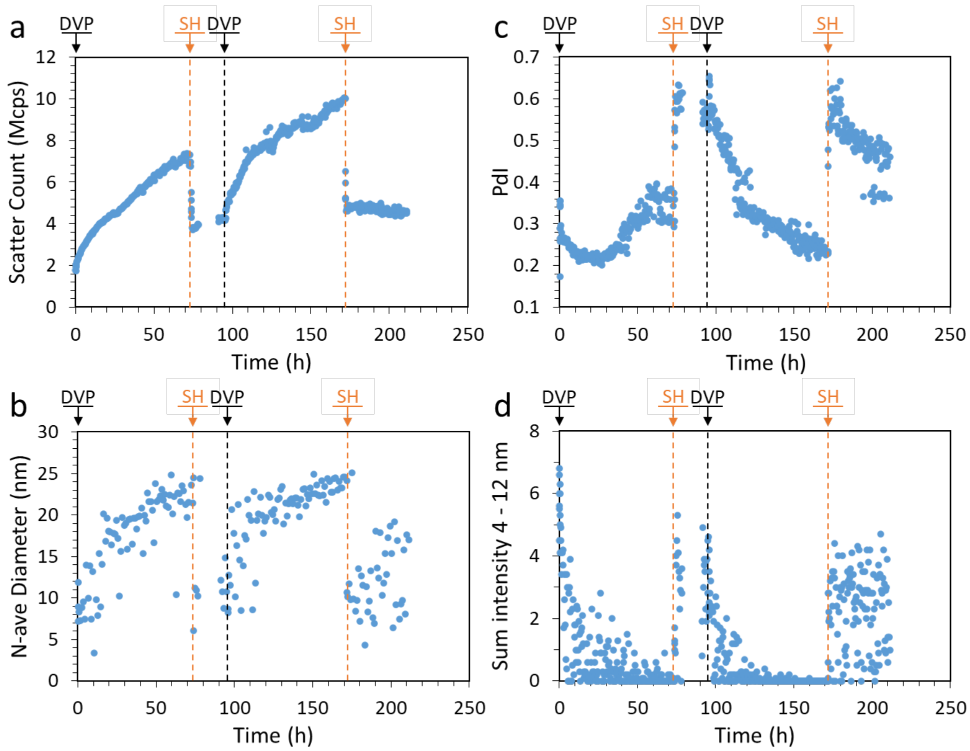

Figure S16. Additional DLS data for DVP and thiol (SH) signal induced C3M assembly and disassembly experiment at 37.5°C (See Figure 3, [P1] = [PSS] = 4 mM; DVP and SH additions at 1.0 eq). a) Normalised scatter count, b) number average diameter, c) PdI and d) sum of intensity plot values between 4 and 12 nm, corresponding with the size range exclusively observed for unimers.

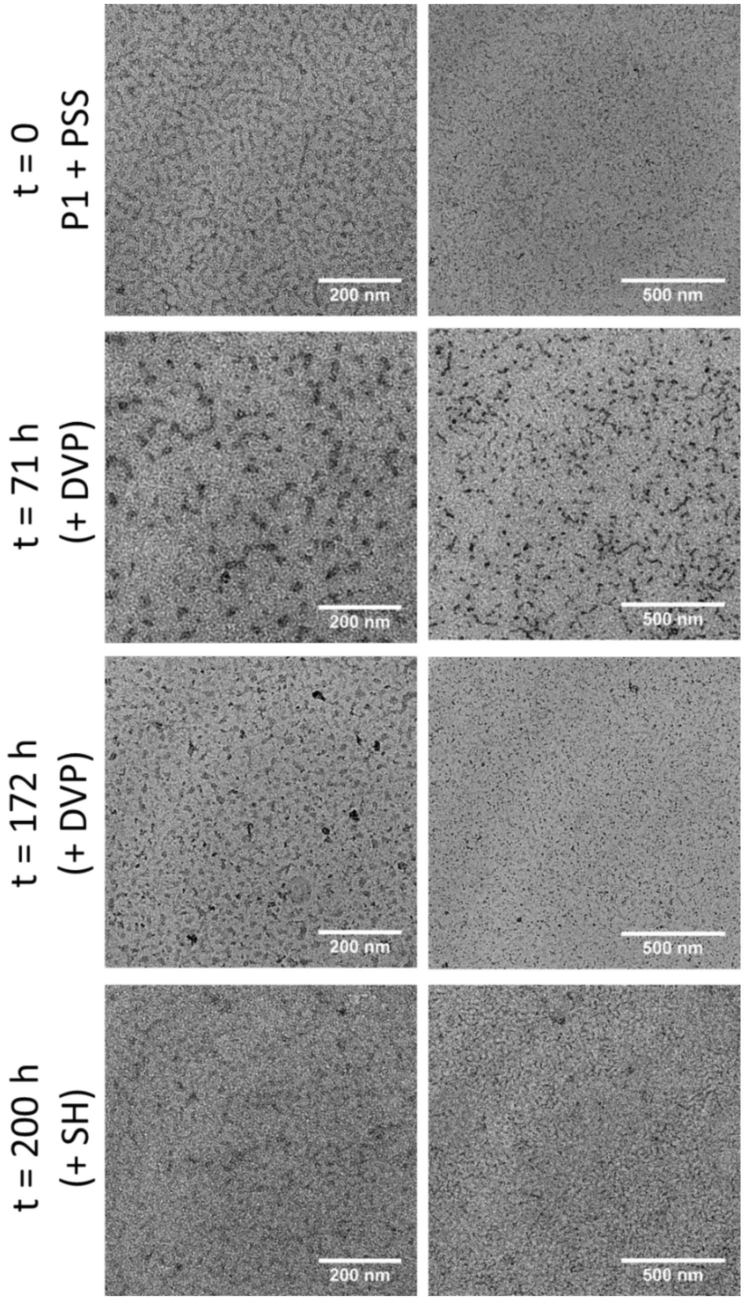

Figure S17. Additional TEM images of samples taken at the end point of each addition during sequential for DVP and thiol (SH) signal induced C3M assembly and disassembly experiment at 37.5°C (See Figure 3, [P1] = [PSS] = 4 mM; DVP and SH additions at 1.0 eq). Note that data for t = 95 h (+SH) is missing due to a sampling error (sample not taken at this time point). Micelle structures at t = 71 and 172 h were measured to be 23.7 ± 3.6 nm and 14.0 ± 3.4 nm in general agreement with DLS data.

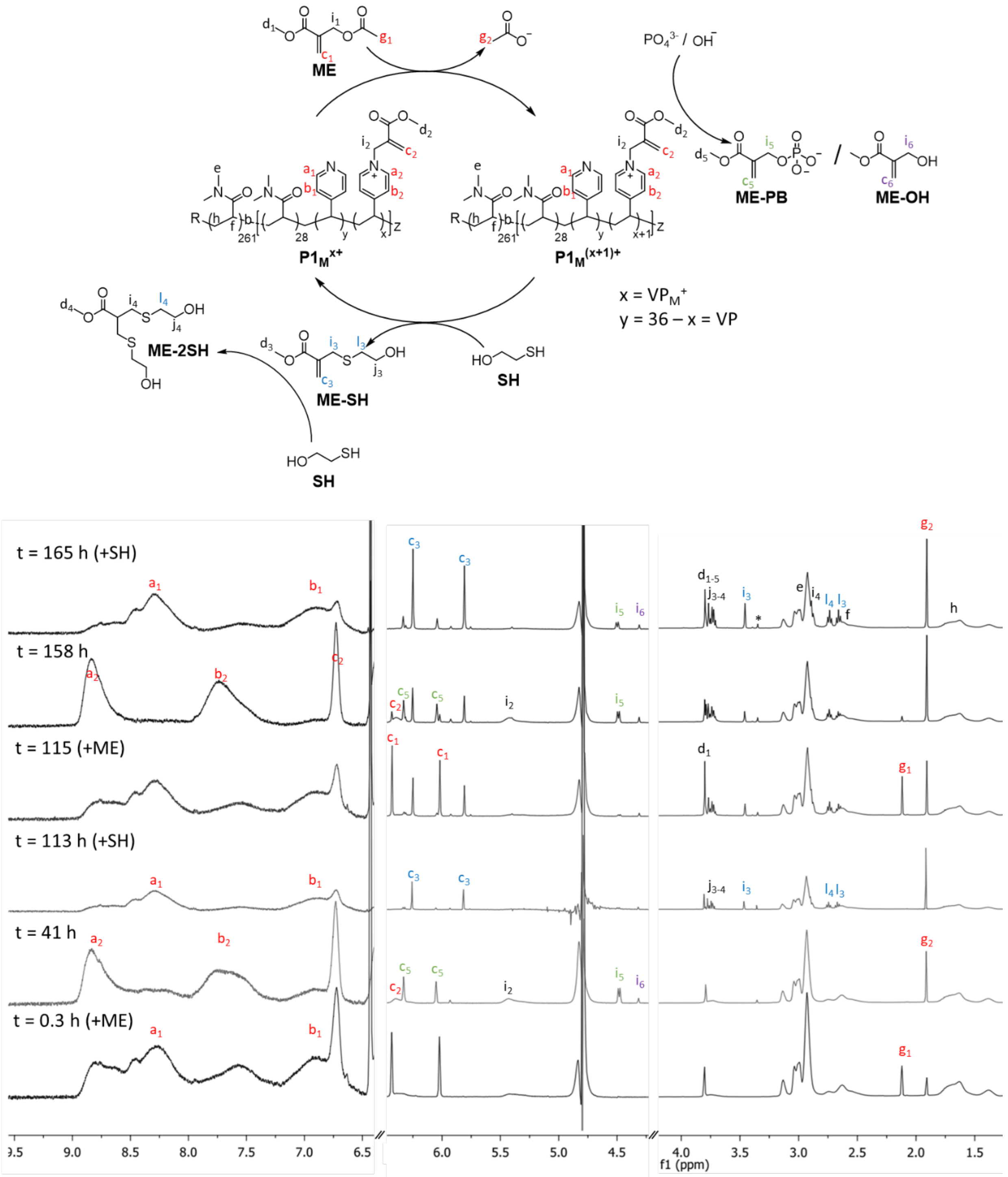

Figure S18. Example ^{1}H NMR (D_2O, pre-sat) spectra from ME and SH signal induced P1 (de)ionization experiment (See Figure 4, [P1] = 20 mM; ME and SH additions at 1.0 eq). Peaks labelled in red and blue were integrated to quantitate the extent of conversion to cationic pyridine adduct (VP_M^+) in P1 and waste (ME-SH and ME-2SH), respectively. Additional allylic species

were identified during the reaction (ME-PB and ME-OH), believed to be the phosphate substitution and hydrolysis product of VP_M^+ (see Figure S20 for further discussion and evidence). Note that the spectra are cut into 3 rescaled segments for clarity. Polyanions excluded from this experiment to avoid supression of signals in micelles. The signal marked with (*) is believed to be due to methanol, which could arise from ester hydrolysis of many species in this reaction network.

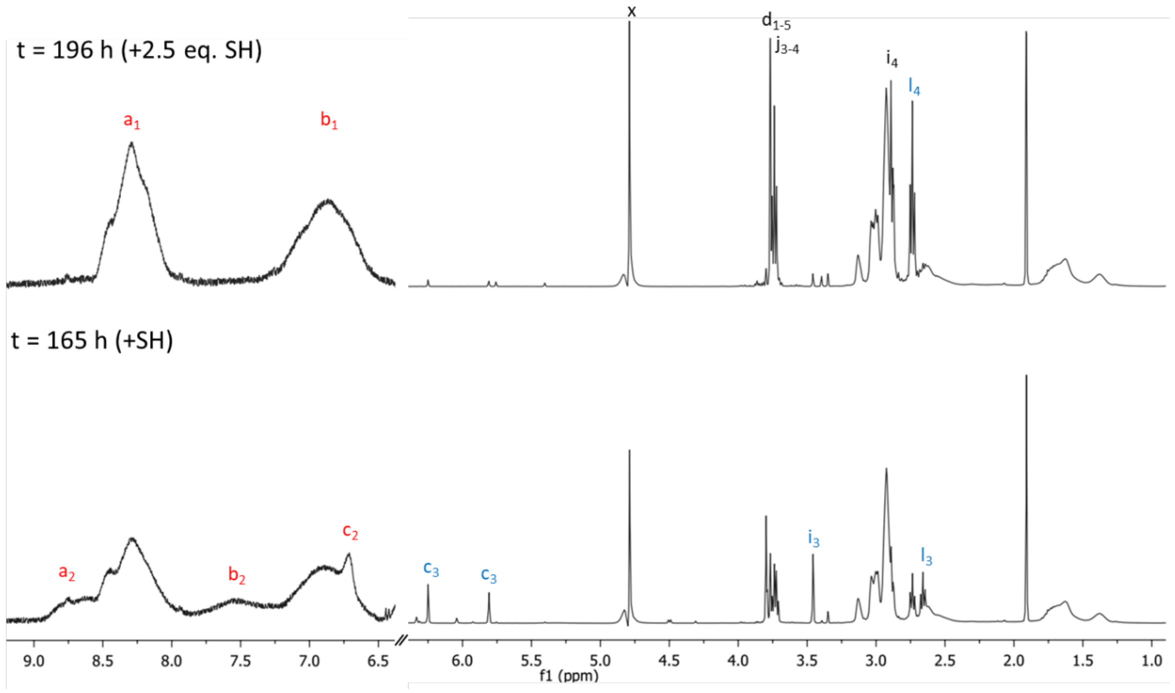

Figure S19. Additional ^{1}H NMR (D_2O, pre-sat) spectra from ME and SH signal induced P1 (de)ionization experiment (See Figure 4 and Figure S18). After addition of a further SH (up to ~ 2.5 equivalents), complete reformation of the starting polyamine is demonstrated. The additional SH is required due to double Michael adduct (ME-2SH) formation, which after addition of excess SH is the main waste product (insignificant ME-SH remaining from ^{1}H NMR). Note that spectra are cut into 2 rescaled segments for clarity.

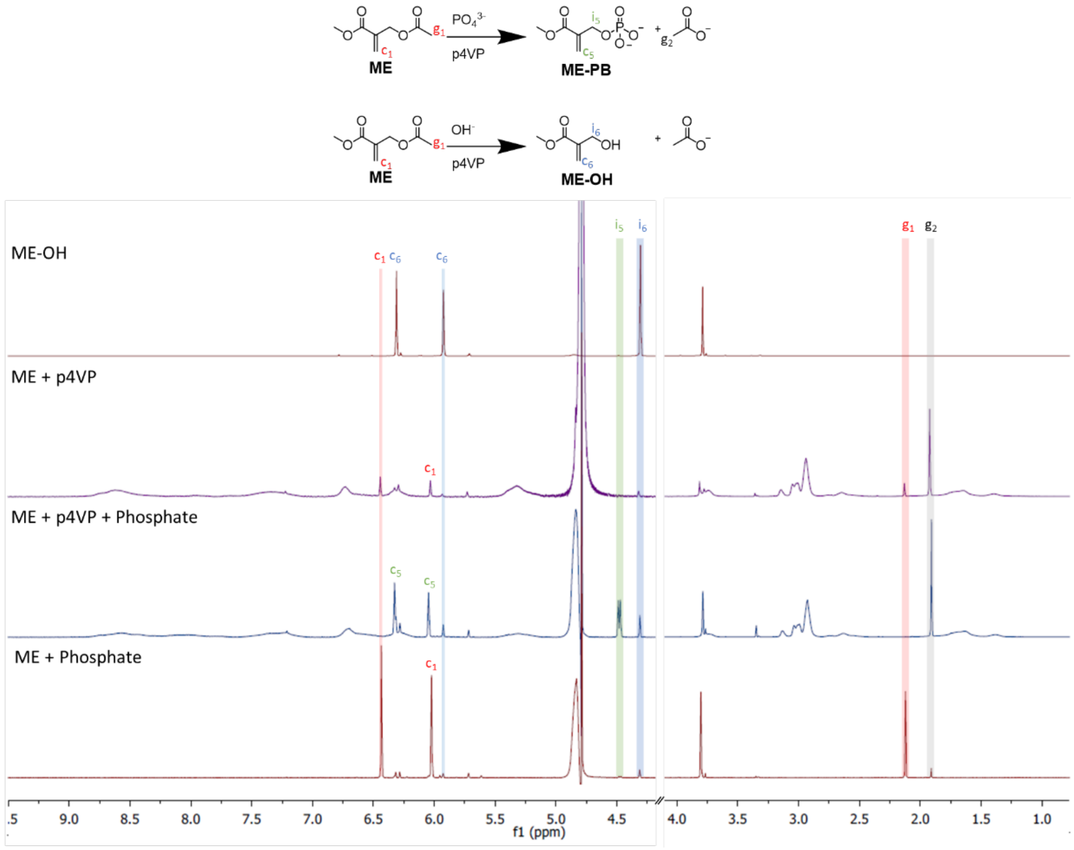

Figure S20. ^{1}H NMR (D_2O, + 100 mM PB buffer pH 7.4 where described) study of additional allylic species formed during ME fuelled P1 ionization. Additional species are proposed to be the phosphate substitution product (ME-PB) and the hydrolysis product (methyl 2-(hydroxymethyl)acrylate), ME-OH). a) ME-OH is commercially available (Fluro Chem) and a reference spectrum is presented. b) ME was combined with a polymer similar to P1 (p4VP$_{104}$-b-DMA$_{261}$) in the absence of PB buffer, spectra shown is after 120 h. c) ME was combined with p4VP$_{104}$-b-DMA$_{261}$ in PB buffer (30% D_2O), spectra shown is after 120 h. d) ME was dissolved into PB buffer (30% D_2O) alone, spectra shown is after 160 h. In all cases some hydrolysis was observed (3 – 7% of ME after > 100 h). However only in cases where PB buffer was included is the doublet at 4.48 ppm observed (J= 6.5 Hz), along with allylic peaks at 6.33 and 6.05 ppm (shifted slightly from other allylic peaks). These peaks are proposed to correspond therefore to the phosphate substituted product (ME-PB), where the splitting at 4.48 ppm is in agreement with literature values for similar allyl phosphates (2-Methyl-2-propenyl 1 Dihydrogen Phosphate).[4] Of note is the apparent catalytic behaviour of p4VP for ME-PB formation, where with only phosphate conversion is limited to ~2% after 120 h, while with p4VP ~25% conversion after 20 h is reached.

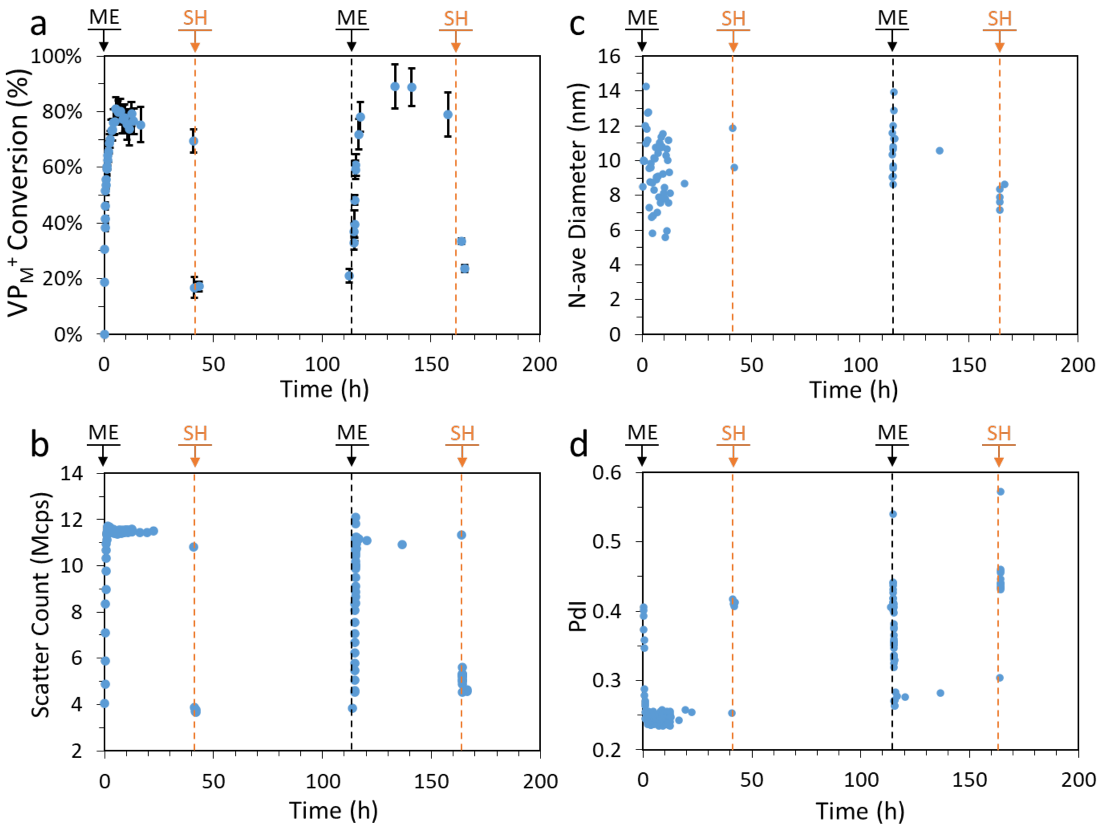

Figure S21. Additional DLS and NMR data for high P1 concentration ME and SH signal induced C3M assembly and disassembly experiment (See Figure 4, [P1] = 20 mM; ME and SH additions at 1.0 eq). For all except a) (NMR experiment) [PSS] = 20 mM. The conversion values reported in a) are as reported in Figure 4a which are an average from integration of shifting aromatic, ME allyl and acetate CH_3 peaks with error bars ± SD of these different measurements. The less clear trends observed in N-ave diameter are attributed to issues arising from high polymer concentration (possibly multiple scattering).

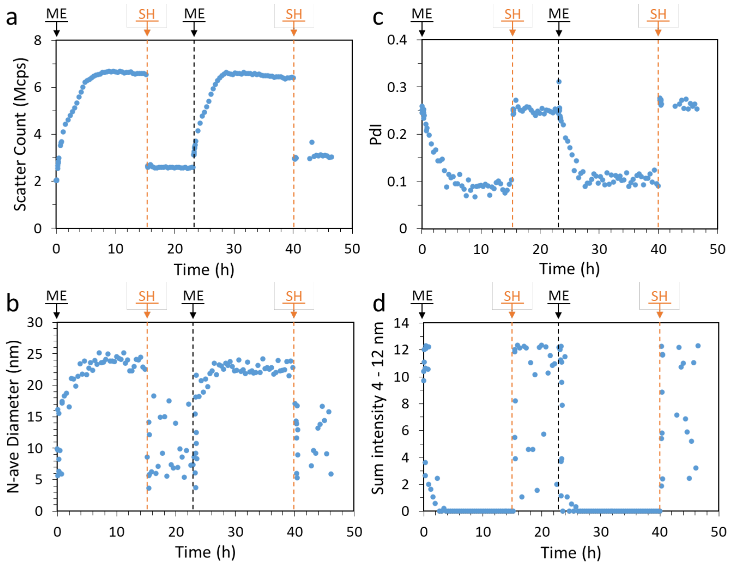

Figure S22. Additional DLS data for low P1 concentration ME and SH signal induced C3M assembly and disassembly experiment (See Figure 4, [P1] = [PSS] = 4 mM; ME and SH additions at 1.0 eq). a) Normalised scatter count, b) number average diameter, c) PdI and d) sum of intensity plot values between 4 and 12 nm, corresponding with the size range exclusively observed for unimers.

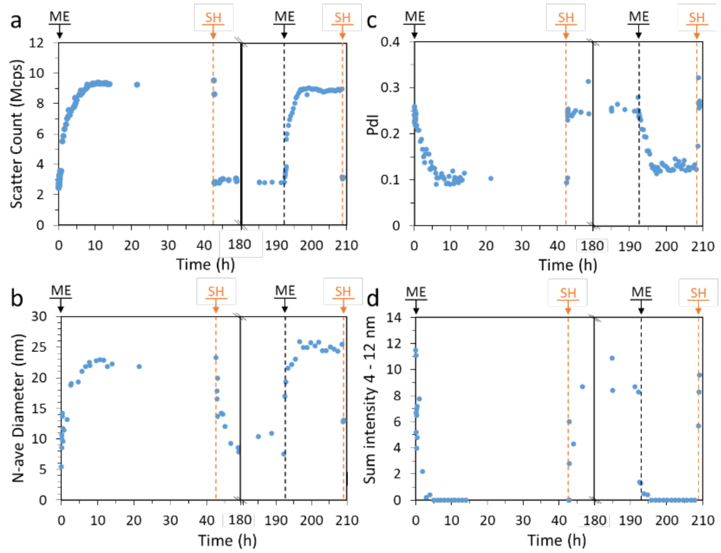

Figure S23. Repeat experimental data for ME and SH signal induced C3M assembly and disassembly experiment ([P1] = [PSS] = 4 mM; ME and SH additions at 1.0 eq). The data presented is for a repeat run with larger time gaps between additions than that shown in the main text, demonstrating excellent reproducibility of the system.

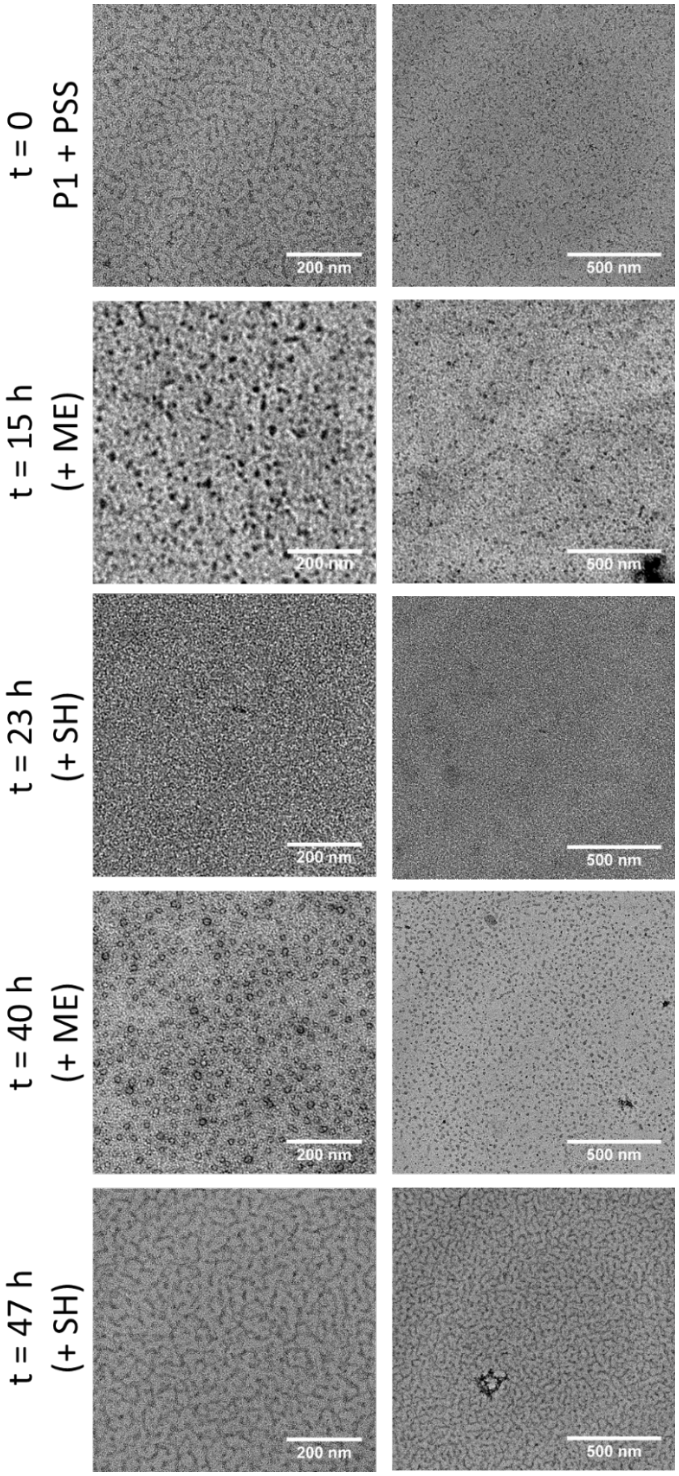

Figure S24. Additional TEM images of samples taken at the end point of each addition during sequential ME and SH signal induced C3M assembly and disassembly experiment (see Figure 4, [P1] = [PSS] = 4 mM; ME and SH additions at 1.0 eq). Micelle structures at t = 15 and 40 h were measured to be 23.3 ± 4.4 nm and 17.8 ± 2.6 nm in agreement with DLS data.

Transient C3M disassembly

P1 (7.3 mg, 8.0 µmol amine), PSS (1.65 mg, 8.0 µmol sulfonate) and 100 mM PB pH 7.4 buffer (2.0 mL, ~25% D_2O) were combined and filtered (0.45 µm filter). DVP (5.7 mg, 24 µmol) was then added to the polymer solution, which was split into two 1.0 mL samples for DLS and ^{1}H NMR monitoring. Once micelle assembly appeared to be complete, a sample was taken for TEM and SH (1.0 eq.) was added to both the DLS and ^{1}H NMR sample. Further additions of SH (1.0 eq.) were added at later time points once the system appeared to return to near equilibrium. For experiments with ME, the same procedure was followed with DVP replaced with ME (3.8 mg, 24 µmol). For select ^{1}H NMR spectra, DLS data and TEM images from these experiments see **Figures S25 – S29**.

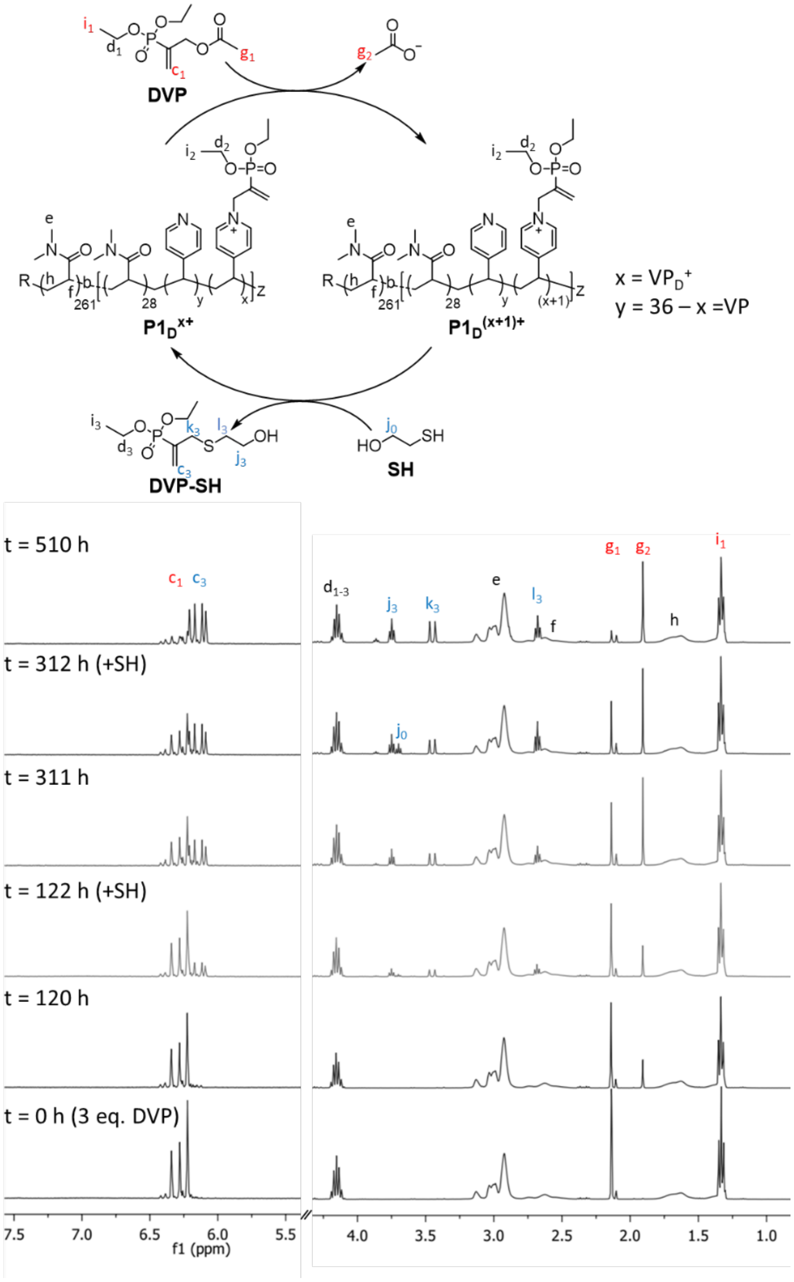

Figure S25. Example ^{1}H NMR (D_2O, pre-sat) spectra from SH (1 eq.) induced transient C3M disassembly experiment starting with DVP (3.0 eq.) (Figure 5, [P1] = [PSS] = 4 mM, [DVP] = 12 mM). Peaks labelled in red and blue were integrated to quantitate the extent of conversion to cationic pyridine adduct (VP_D^+) in P1 and waste (DVP-SH), respectively. Addition of PSS in experiment lead to supression of peaks related to VP_M^+ due to inclusion in micelle core. Dilute nature of experiment rendered aromatic peaks from P1 poorly resolvable and were thus excluded from analysis. Note that spectra is cut into 2 rescaled segments for clarity.

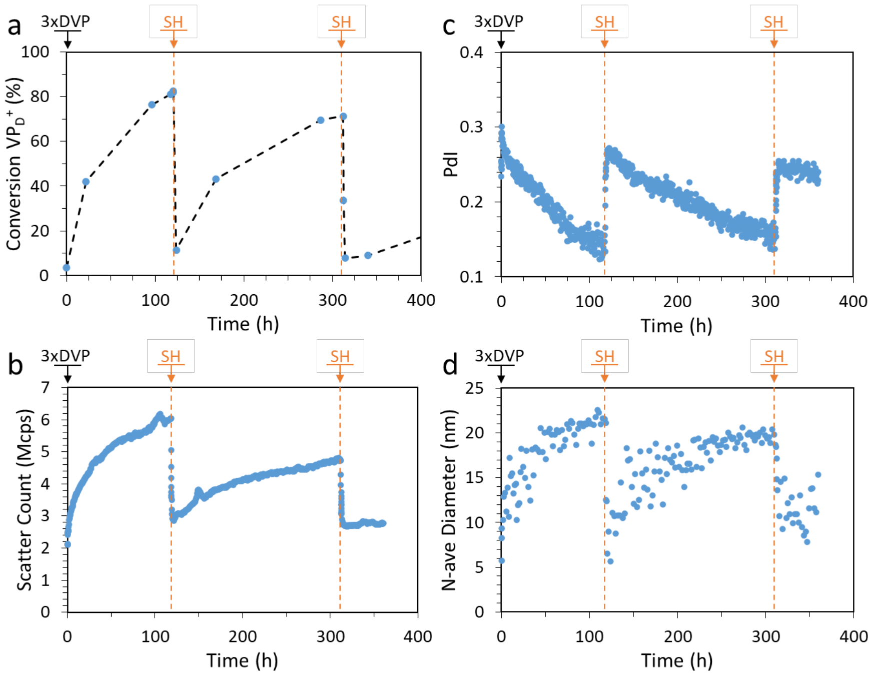

Figure S26. Additional DLS and NMR data for SH (1 eq.) induced transient C3M disassembly experiment starting with DVP (3.0 eq.) (Figure 5, [P1] = [PSS] = 4 mM, [DVP] = 12 mM). Note NMR data presented in a) for conversion to VP_D^+ is an estimate obtained by assuming the difference between DVP consumed and DVP-SH produced equates to VP_D^+ production since the signals for the species are supressed in the micelle core. Value is expressed as theoretical % of VP units on P1 converted to VP_D^+.

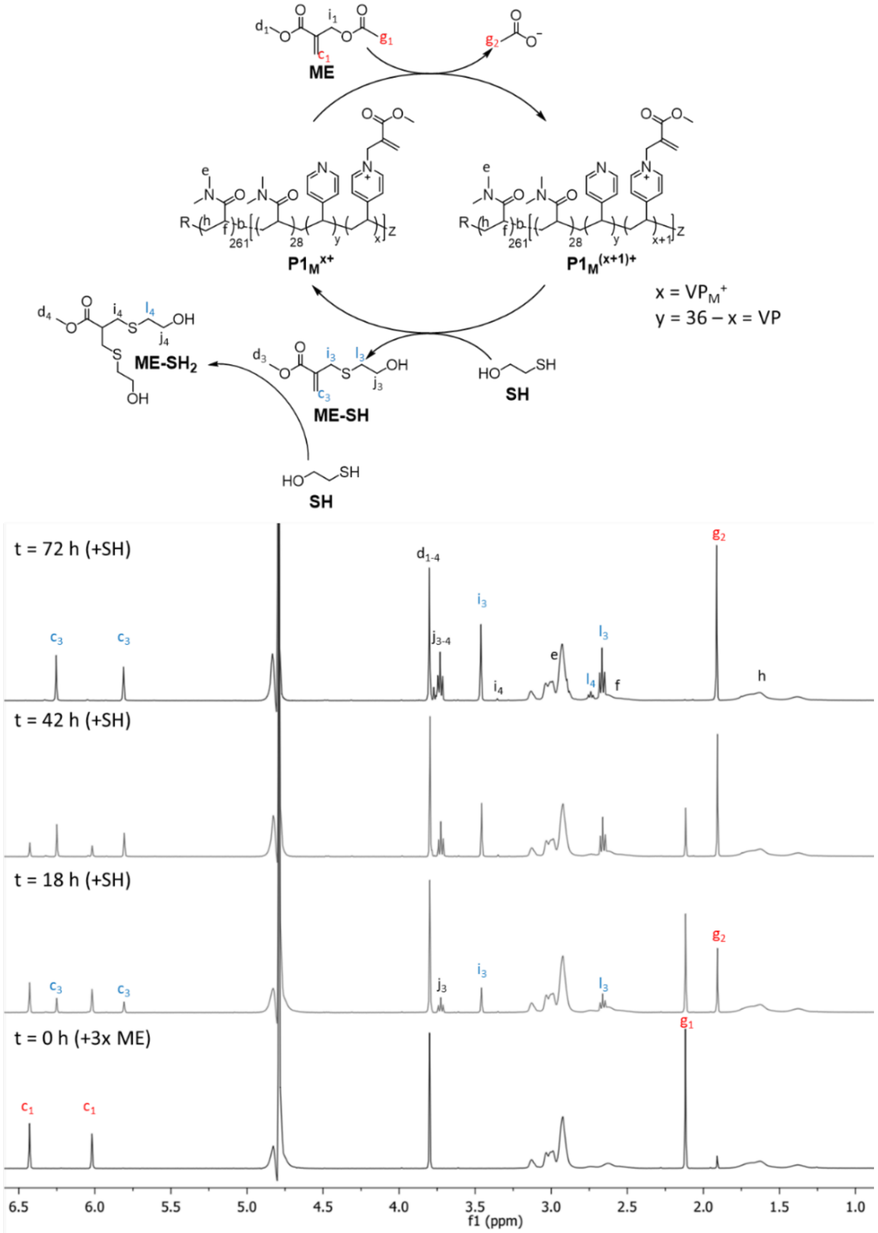

Figure S27. Example ^{1}H NMR (D_2O, pre-sat) spectra from SH (1.0 eq.) induced transient C3M disassembly experiment starting with ME (3.0 eq.) (Figure 5, [P1] = [PSS] = 4 mM, [ME] = 12 mM). Peaks labelled in red and blue were integrated to quantitate the extent of conversion to cationic pyridine adduct (VP_M^+) in P1 and waste (ME-SH and ME-SH_2), respectively. Addition of PSS in experiment lead to supression of peaks related to VP_M^+ due to inclusion in micelle core. Dilute nature of experiment rendered aromatic peaks from P1 poorly resolvable and were thus excluded from analysis.

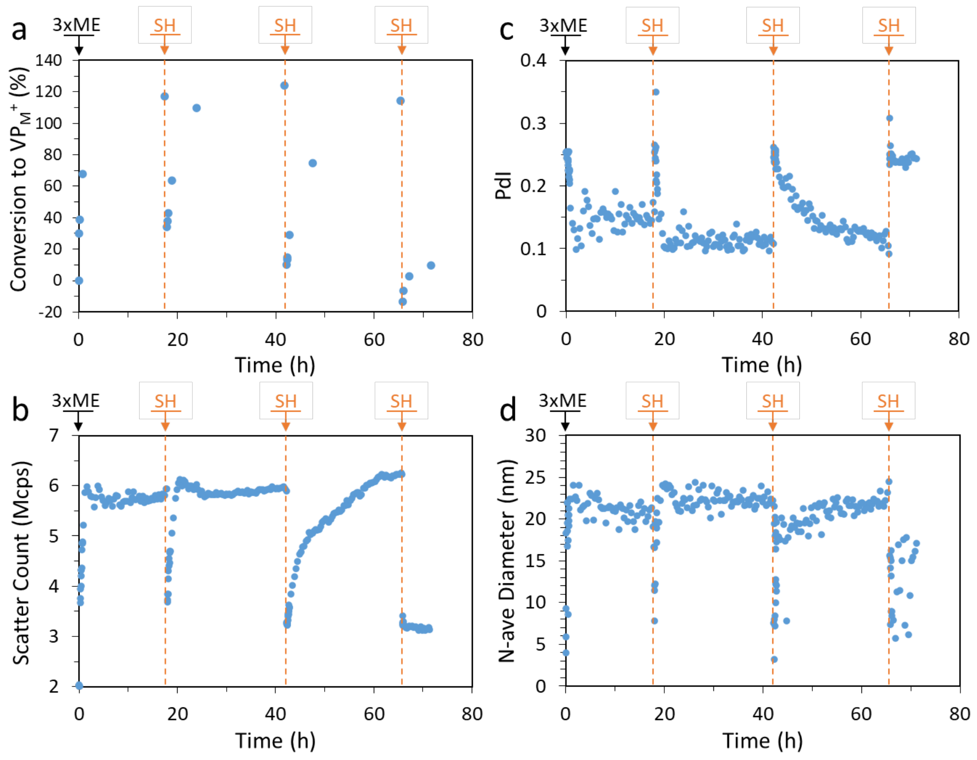

Figure S28. Additional DLS and NMR data for SH (1 eq.) induced transient C3M disassembly experiment starting with ME (3.0 eq.) (Figure 5, [P1] = [PSS] = 4 mM, [ME] = 12 mM). Note NMR data presented in a) for conversion to VP_M^+ is only an estimate obtained by assuming the difference between ME consumed and ME-SH + ME-SH_2 produced equates to VP_M^+ production since the signals for the species are supressed in the micelle core. Value is expressed as theoretical % of VP units on P1 converted to VP_M^+.

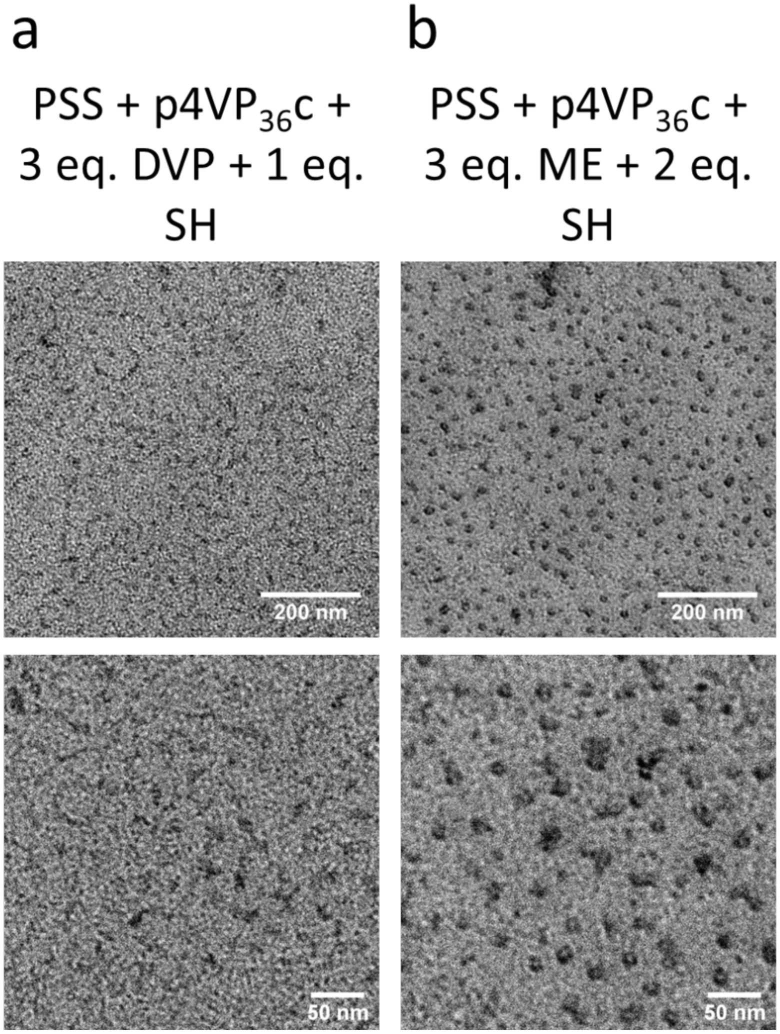

Figure S29 TEM images demonstrating extent of micelle re-assembly in transient C3M disassembly experiments (starting with 3 eq. of an allyl acetate) after 2.0 eq. of SH have been added a) with DVP as the allyl acetate micelle re-assembly appeared to be incomplete, even after at t = 1000 h. Spherical objects were measured as 13.8 ± 4.9 nm. b) with ME as the allyl acetate micelle assembly appeared to be successful at t = 65 h. Spherical objects were measured as 17.5 ± 2.7 nm.

Fuel-driven transient C3M assembly

P1 (5.5 mg, 6.0 μmol amine), PSS (1.24 mg, 6.0 μmol sulfonate), Thr (5.7 mg, 48 μmol) and 0.25 M PB pH 7.4 buffer (1.5 mL, ~25% D_2O) were combined and filtered (0.45 μm filter). After recording baseline data, 100 μL was taken for an initial TEM measurement. Then DVP (2.8 mg, 12 μmol) was added to the remaining solution, which was split into two 0.7 mL samples for DLS and ^{1}H NMR monitoring. Once micelle assembly appeared to peak (t = 85 h), a 100 μL sample was taken for TEM from the DLS sample. Analysis continued until 500 h where micelle disassembly appeared to be complete. For experiments with ME, the same procedure was followed with DVP replaced with ME (1.9 mg, 12 μmol) and Thr addition was reduced (3.6 mg, 30 mmol). For both fuels an additional two matching DLS experiments were later conducted, allowing for the DLS data to be recorded in triplicate. Values are reported as the mean from the 3 measurements, with selected error bars representing SD at that time range. The data was fit with a two exponential function (see **Equation S4**), as can be crudely derived for the system if both forward and back reactions are assumed to be pseudo first order reactions. For select ^{1}H NMR spectra, additional DLS data and TEM images from these experiments see **Figures S30 – S37**.

$$y = ae^{-bx} + ce^{-dx} + f \quad \textbf{Eq. S4}$$

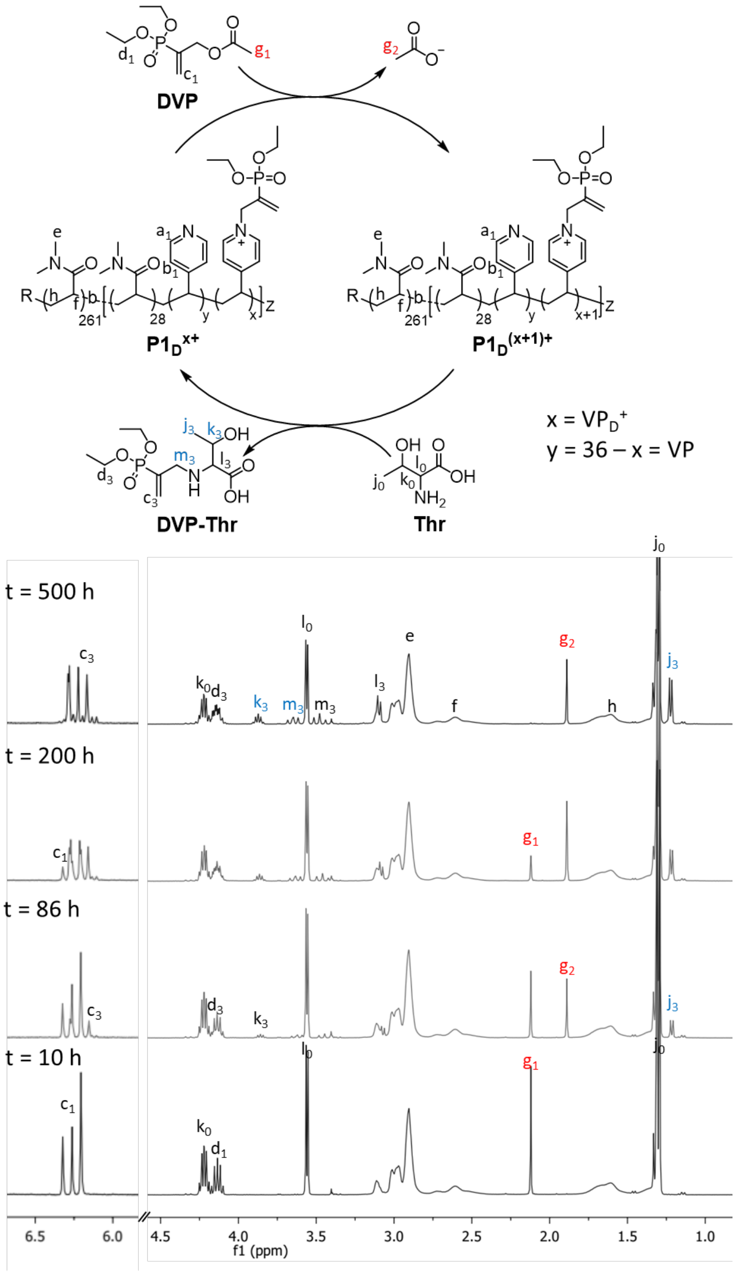

Figure S30. Example ^{1}H NMR (D_2O, pre-sat) spectra from DVP fuelled (2.0 eq.) transient C3M sassembly (Figure 6, [P1] = [PSS] = 4 mM, [Thr] = 32 mM, [DVP] = 8 mM (addition at 10 h)). Peaks labelled in red and blue were integrated to quantitate the extent of conversion to cationic pyridine adduct (VP_D^+) in P1 and waste (DVP-Thr), respectively. Addition of PSS in experiment lead to supression of peaks related to VP_D^+ due to inclusion in micelle core. Note that DVP-Thr contains a secondary amine known to also react with VP_D^+ and although not identified here is likely also have formed.[5] Spectra is cut into 2 rescaled segments for clarity.

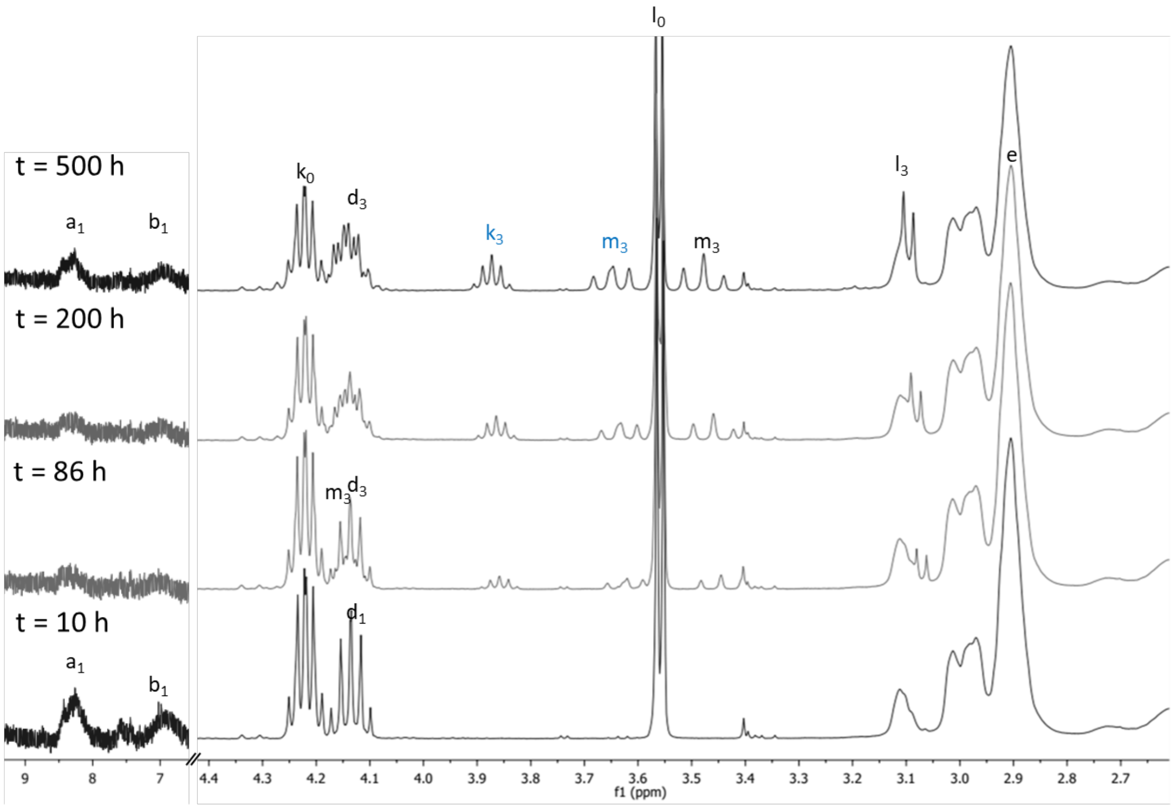

Figure S31. Close up ^{1}H NMR (D_2O, pre-sat) spectra from 2.6 to 4.4 ppm and 6.5 to 9.5 ppm from DVP fuelled (2.0 eq.) transient C3M assembly (Figure 6, [P1] = [PSS] = 4 mM, [Thr] = 32 mM, [DVP] = 8 mM (addition at 10 h)). Note that spectra is cut into 2 rescaled segments for clarity.

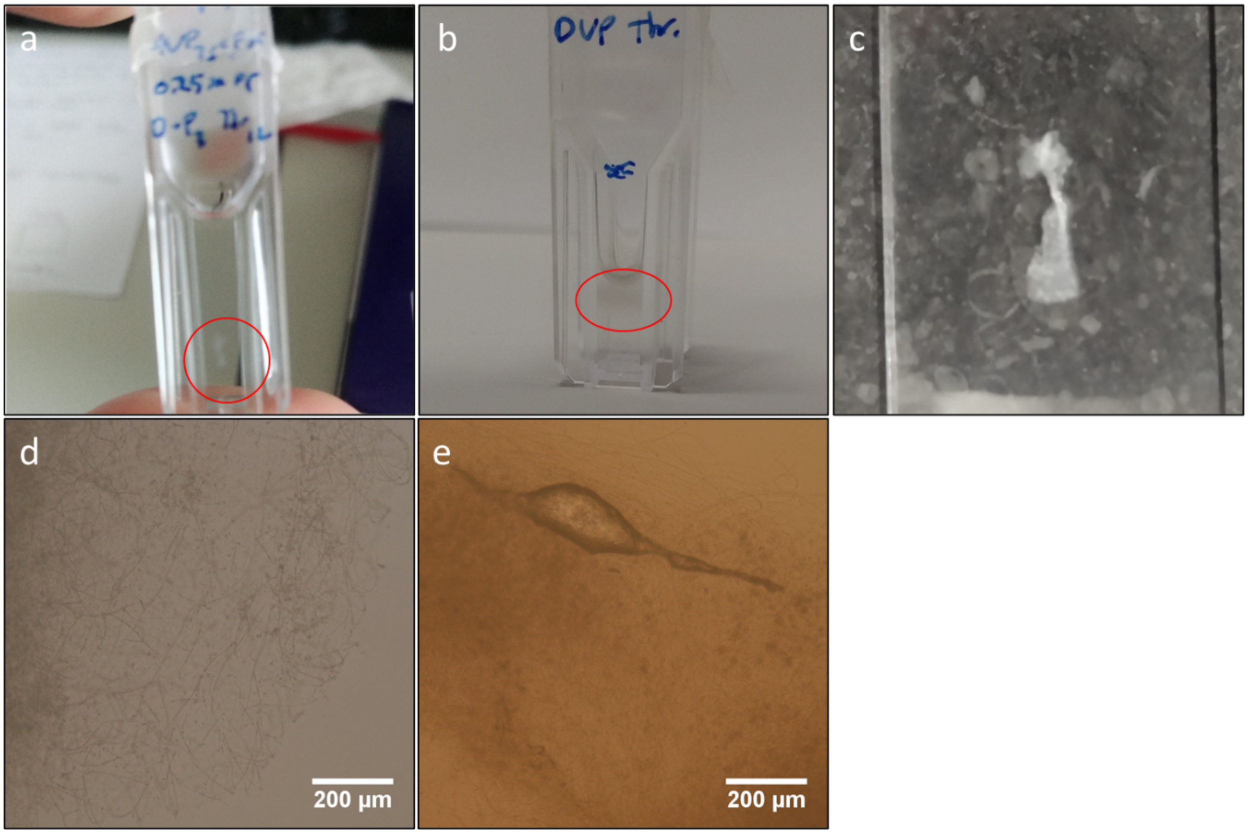

Figure S32. Images of proposed ‘biological growth’ (likely bacterial contamination) from DVP fuelled transient C3M assembly experiment (Figure 6, [P1] = [PSS] = 4 mM, [Thr] = 32 mM, [DVP] = 8 mM (addition at 10 h)). Between t = 250 h and 300 h an opaque filamentous substance appeared in all 3 samples. Due to its interefence with DLS measurements (see Figure S43) each sample was filtered through a 0.45 µm filter, with the filtered material stored for later identification. a) Unidentified ‘biological growth’ suspended in solution in DLS cuvette before filtering. b) After filtering samples, the filtered material was separated and stored where it continued to grow. c) Image of ‘biological growth’ on optical microscope slides, material was opaque, soft and strechy. d-e) Optical microscopy images of proposed ‘biological growth’, clear filamentous structures can be observed indicating species is likely of biological origin rather than a precipitate from the ongoing chemcial reaction network.

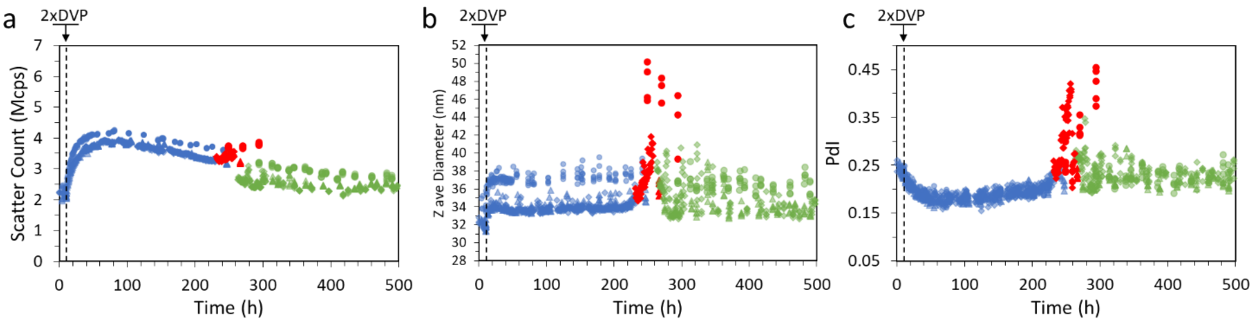

Figure S33. Comparison of DLS data before and after filtering for DVP fuelled transient C3M assembly experiment (Figure 6, [P1] = [PSS] = 4 mM, [Thr] = 32 mM, [DVP] = 8 mM (addition at 10 h), data from triplicate experiment). Data points are colour coded as blue (before filtering), red (before filtering and removed from analysis due to interference from 'biological growth') and green (after filtering). The filtering process may have artificially reduced scatter count by removing C3Ms, however considering filtering was conducted with 0.45 µm filter and the C3Ms are measured as <0.1 µm the effect is expected to be minimal. Figure demonstrates that the a) scatter count, b) Z-ave diameter and c) PdI continue on trend after filtering.

In similar experiments in our laboratory we have found that addition of a biocide such as NaN_3 (0.02 wt%) at the start of the experiment can avoid the formation of this growth. Note that due the strong nucelophilicy of NaN_3 additional fuel may be required to compensate.

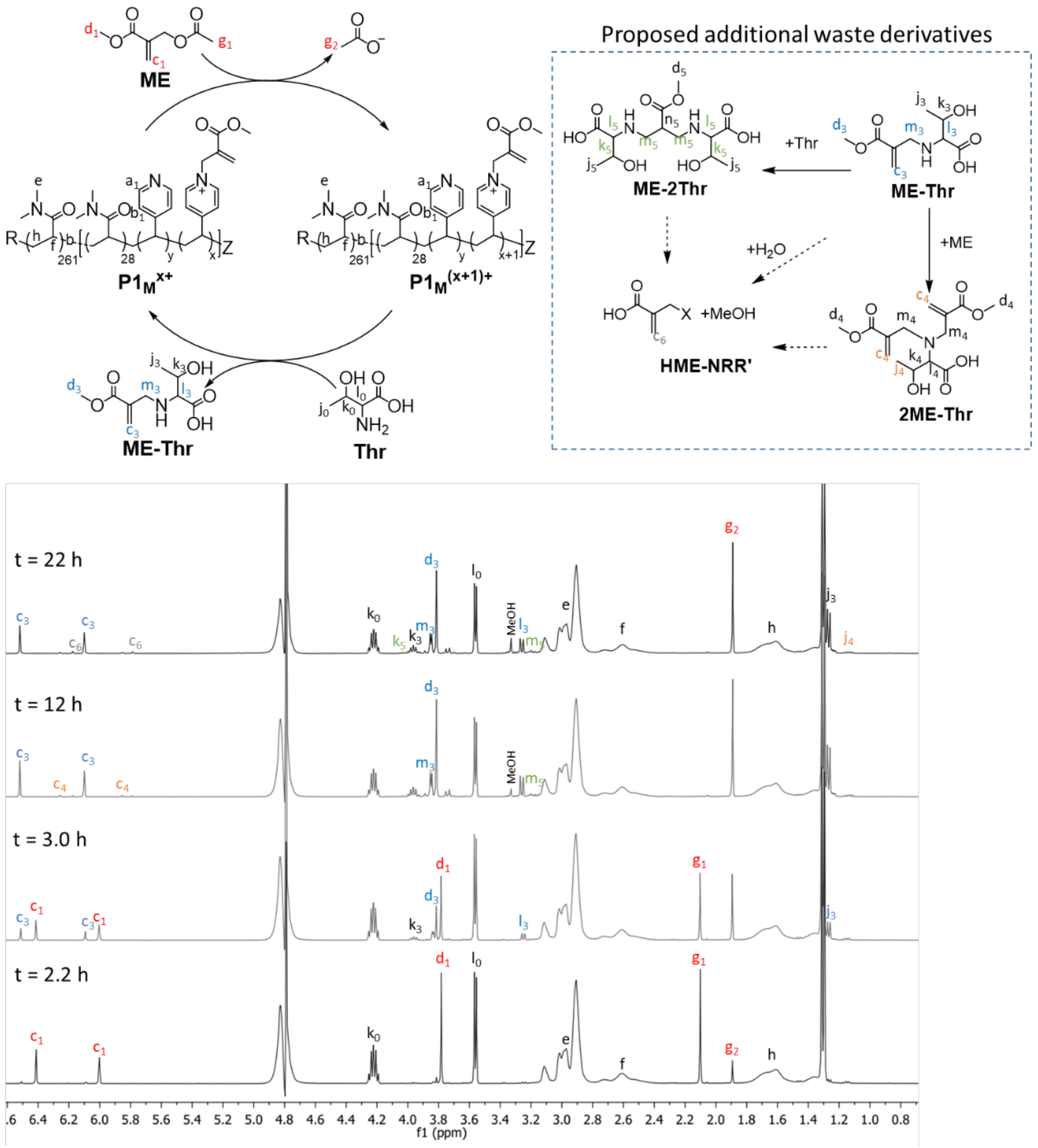

Figure S34. Example ^{1}H NMR (D_2O, pre-sat) spectra from ME fuelled (2.0 eq.) transient C3M assembly (Figure 6, [P1] = [PSS] = 4 mM, [Thr] = 20 mM, [ME] = 8 mM (addition at 2 h)). Peaks labelled in red, blue, green, organge and grey were integrated to quantitate the extent of conversion to cationic pyridine adduct (VP_M^+) in P1 and wastes (ME-Thr, 2ME-Thr, ME-2Thr and ester hydrolysed dervatives (HME-NRR')), respectively. These waste structures are merely proposed structures, however their use does allow for near complete (within 5%) account of ME fuel consumption by the end of reaction. Addition of PSS in experiment lead to supression of peaks related to VP_M^+ due to inclusion in micelle core.

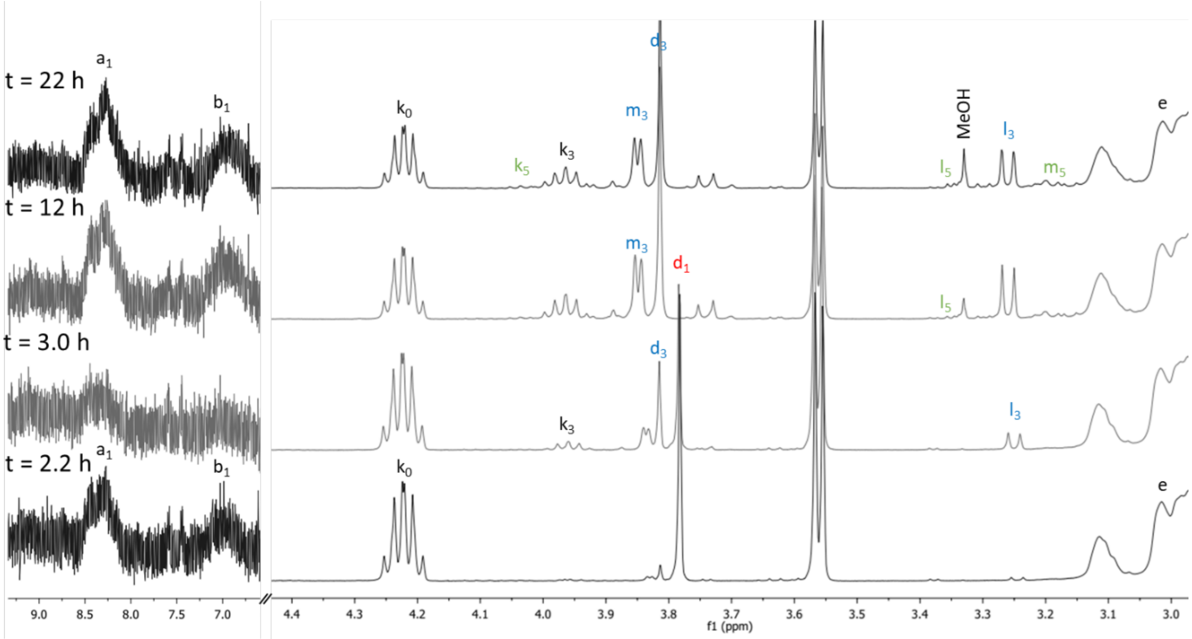

Figure S35. Close up ^{1}H NMR (D_2O, pre-sat) spectra between 9.5 and 6.5 ppm (demonstrating suppresion / loss of aromatic VP signals during C3M assembly) and 4.4 ppm to 3.0 ppm. Spectra are from ME fuelled (2.0 eq.) transient C3M assembly (Figure 6, [P1] = [PSS] = 4 mM, [Thr] = 20 mM, [ME] = 8 mM (addition at 2 h)). Note that spectra is cut into 2 rescaled segments for clarity.

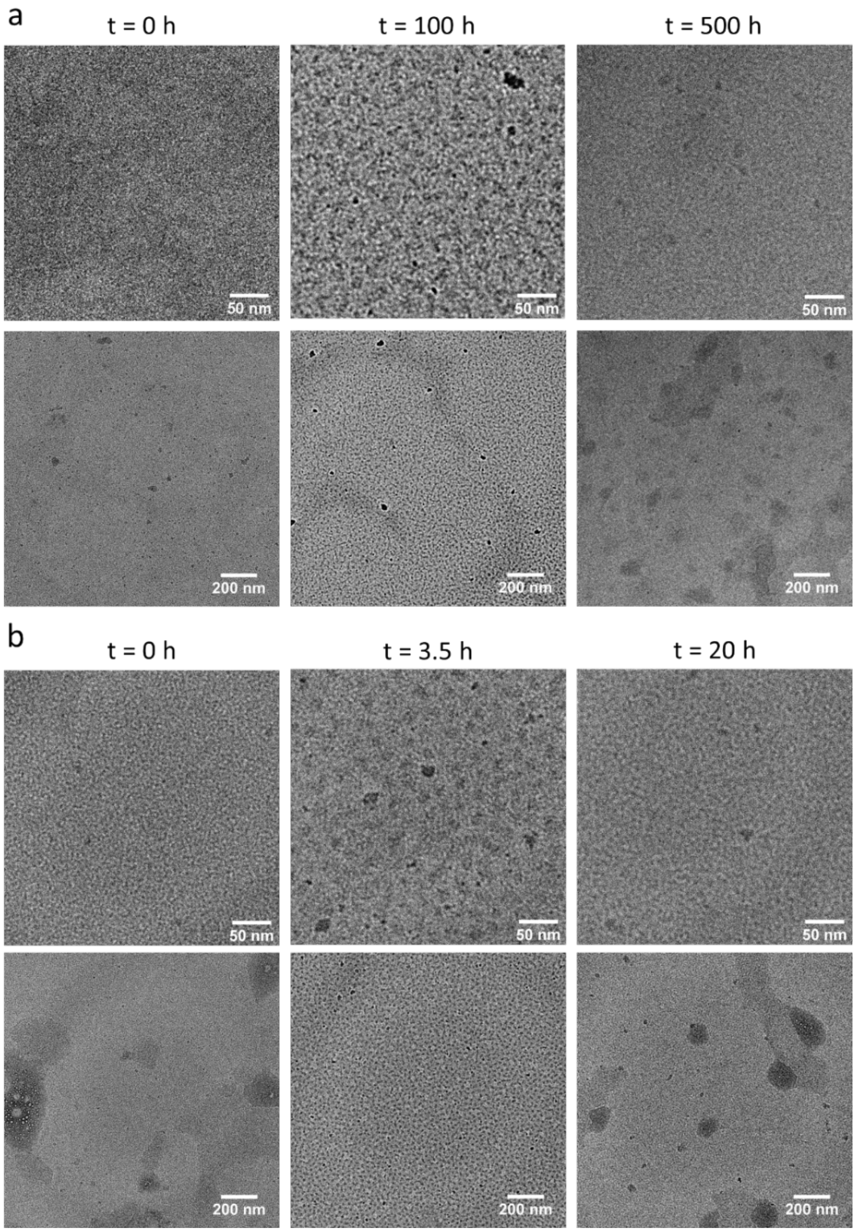

Figure S36. Representative TEM images (without uranyl acetate stain) from fuel-driven transient C3M assembly experiments. a) With DVP as the fuel where the spherical objects at t = 100 h were measured to be 12.8 ± 4.7 nm. b) With ME as the fuel where the spherical objects at t = 3.5 h were measured to be 16.2 ± 3.4 nm. These images demonstrate a lack of micelle structures present at the start (before addition of fuel) and end (after fuel is depleted). At the peak times spherical micelle like structures are present. The absence of such structures in the stained samples (**Figure S37**) may be that (due to lower extent of P1 ionization compared to other experiments) cationic uranyl ions lead to micelle destruction during sample preparation phase.

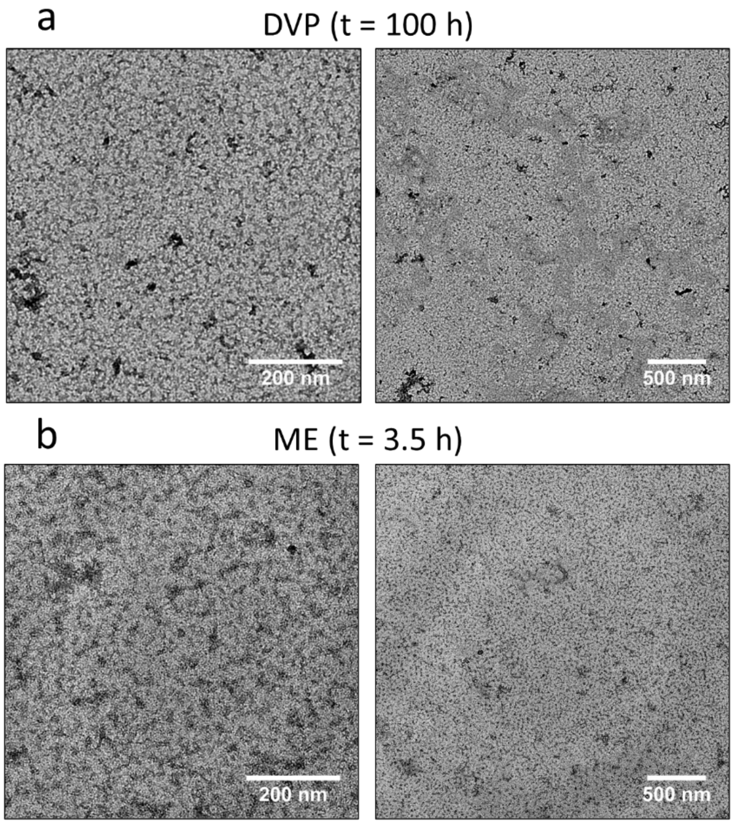

Figure S37. Reference uranyl acetate-stained TEM images from fuel-driven transient C3M assembly experiments taken at peak state of assembly according to DLS data. a) With DVP as the fuel (t = 100 h) and b) with ME as the fuel (t = 3.5 h). While micelle structures are not clearly present in these images, while without a stain (**Figure S36**) we were able to visualise micelle structures.

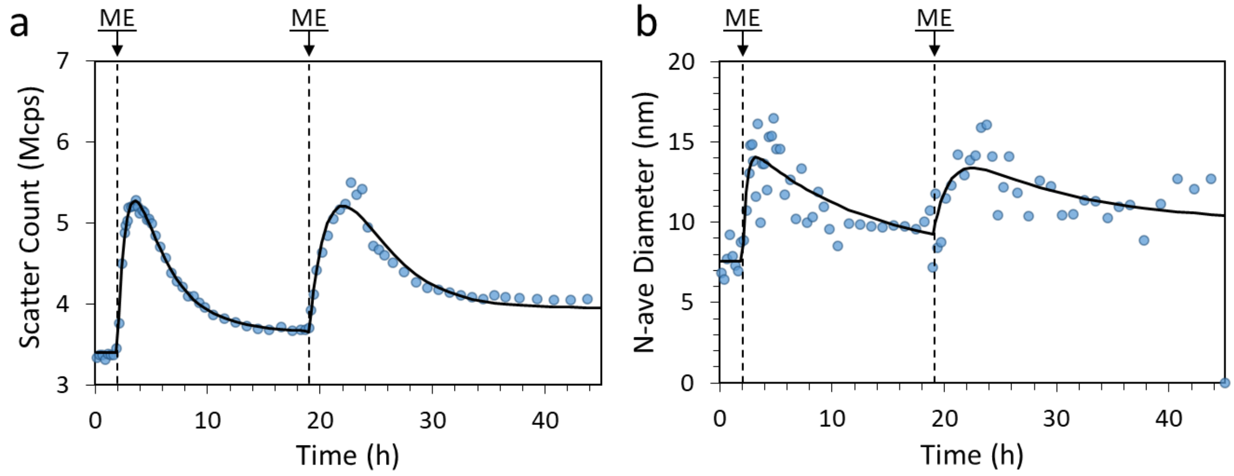

Figure S38. Two cycle fuel-driven transient C3M assembly by successive additions of fuel (ME, 2x6 mM, 1.5 eq. each) at 2 and 19 h to a solution of P1 (4 mM), PSS (4 mM) and Thr (24 mM). Solutions were maintained at pH 7.4 by 250 mM PB buffer. (a) Normalised scatter count of sample as measured by DLS and (b) number average diameter as measured by DLS. Two-exponential curves are fitted after each ME addition to guide the eye.

References

1. C. Garzon, M. Attolini and M. Maffei, *Tetrahedron Lett.*, **2010**, 51, 3772-3774.
2. M. Rambaud, A. d. Vecchio and J. Villieras, *Synth. Commun.*, **1984**, 14, 833-841.
3. M. Nascimento de Oliveira, S. Arseniyadis and J. Cossy, *Chem. Eur. J.*, **2018**, 24, 4810-4814.
4. E. T. Harper, B. H. Phelps and V. F. Riser, *Prep. Biochem.*, **1976**, 6, 347-368.
5. B. Klemm, R. Lewis, I. Piergentili and R. Eelkema, **2021**, ChemRxiv. preprint DOI: 10.33774/chemrxiv-2021-jh5jm
